# No Evidence That Homologs of Key Circadian Clock Genes Direct Circadian Programs of Development or mRNA Abundance in *Verticillium dahliae*

**DOI:** 10.3389/fmicb.2020.01977

**Published:** 2020-08-28

**Authors:** Emma Cascant-Lopez, Susan K. Crosthwaite, Louise J. Johnson, Richard J. Harrison

**Affiliations:** ^1^Genetics, Genomics and Breeding, NIAB EMR, East Malling, United Kingdom; ^2^The School of Biological Sciences, University of Reading, Reading, United Kingdom; ^3^National Institute of Agricultural Botany (NIAB), Cambridge, United Kingdom

**Keywords:** *Verticillium dahliae*, plant pathogen, circadian clock, evolution, gene expression

## Abstract

Many organisms harbor circadian clocks that promote their adaptation to the rhythmic environment. While a broad knowledge of the molecular mechanism of circadian clocks has been gained through the fungal model *Neurospora crassa*, little is known about circadian clocks in other fungi. *N. crassa* belongs to the same class as many important plant pathogens including the vascular wilt fungus *Verticillium dahliae*. We identified homologs of *N. crassa* clock proteins in *V. dahliae*, which showed high conservation in key protein domains. However, no evidence for an endogenous, free-running and entrainable rhythm was observed in the daily formation of conidia and microsclerotia. In *N. crassa* the *frequency* (*frq*) gene encodes a central clock protein expressed rhythmically and in response to light. In contrast, expression of *Vdfrq* is not light-regulated. Temporal gene expression profiling over 48 h in constant darkness and temperature revealed no circadian expression of key clock genes. Furthermore, RNA-seq over a 24 h time-course revealed no robust oscillations of clock-associated transcripts in constant darkness. Comparison of gene expression between wild-type *V. dahliae* and a Δ*Vdfrq* mutant showed that genes involved in metabolism, transport and redox processes are mis-regulated in the absence of *Vdfrq*. In addition, *Vd*Δ*frq* mutants display growth defects and reduced pathogenicity in a strain dependent manner. Our data indicate that if a circadian clock exists in Verticillium, it is based on alternative mechanisms such as post-transcriptional interactions of VdFRQ and the WC proteins or the components of a FRQ-less oscillator. Alternatively, it could be that whilst the original functions of the clock proteins have been maintained, in this species the interactions that generate robust rhythmicity have been lost or are only triggered when specific environmental conditions are met. The presence of conserved clock genes in genomes should not be taken as definitive evidence of circadian function.

## Introduction

Circadian clocks are endogenous timekeepers that enable organisms to anticipate cyclic changes in the environment and thus confer adaptive advantage (Dunlap and Loros, 2006; Baker et al., 2012). The defining characteristics of circadian clocks are: rhythmicity that persists in constant conditions (absent cyclic conditions) with a period of approximately 24 h; the ability to entrain to external signals such as light and temperature; and temperature and nutritional compensation of period (Pittendrigh, 1960). Such oscillations have been widely observed in most branches of life, and are particularly well characterized in *Neurospora crassa*.

In *N. crassa* the circadian clock is based on a transcription-translation negative feedback loop (Dunlap, 1999) initiated by the photoreceptor and transcription factor White Collar-1 (WC-1) (Crosthwaite et al., 1997). In the dark WC-1 and White Collar-2 (WC-2) dimerize through their PAS domains forming the White-Collar Complex (WCC) (Ballario et al., 1998). The WCC binds to the clock-box motif in the promoter of the *frequency* (*frq*) gene, activating its transcription (Froehlich et al., 2002). When FRQ is synthesized it associates with FRQ-interacting RNA helicase (FRH) forming the FRH-FRQ complex (FFC). The FFC inhibits the activity of the WCC, and consequently transcription of *frq* is reduced. FRQ is progressively phosphorylated by the kinase CK1, and degraded by the FWD-1 protein (He et al., 2003). This leads to a reduced FFC-mediated inhibition of the WCC which results in initiation of a new cycle (Dunlap and Loros, 2006). In addition to the FRQ/WC-based oscillator (FWO) described above, *N. crassa* contains other FRQ-independent oscillators known as FRQ-less oscillators (FLOs) (Correa et al., 2003; de Paula et al., 2006; Hunt et al., 2012). For example, the activity of nitrate reductase oscillates in the absence of FRQ (Christensen et al., 2004), and a cryptochrome-dependent oscillator (CDO) (Nsa et al., 2015) has also been described.

Both light and temperature can entrain the clock. Light rapidly induces transcription of *frq*, which leads to clock resetting and entrainment to the external stimulus (Crosthwaite et al., 1995). The WCC complex activates *frq* transcription by binding to light responsive elements (LREs) present in the *frq* promoter (Froehlich et al., 2002). Two LREs have been identified in the *frq* promoter*;* the proximal LRE is necessary for light induction of *frq*, whereas the distal LRE (known as the Clock-Box) is required for both light and circadian clock regulation (Froehlich et al., 2003; Smith et al., 2010). Additionally, the WCC is essential for the transcriptional activation of numerous light-regulated genes, including other transcription factors, leading to a genetic regulatory cascade (Chen et al., 2010). A subset of the downstream genes are known to be regulated by both the circadian clock and light. Included in this subset is the photoreceptor VVD, which is involved in photo-adaptation (Heintzen et al., 2001). Despite not being essential for circadian function, VVD enhances the robustness of the clock by preventing clock resetting at dawn and promoting clock resetting at dusk (Heintzen et al., 2001; Elvin et al., 2005; Chen et al., 2010; Hunt et al., 2010; Malzahn et al., 2010). Other targets include genes that function in carotenoid synthesis and spore development (Smith et al., 2010). Importantly, rhythmic mRNA levels in *N. crassa* are not only controlled by light and the circadian clock at the promoter level, but through post-transcriptional regulation (Hurley et al., 2014). In *N. crassa* the circadian clock regulates approximately 40% of the genome and a quarter of expressed proteins (Hurley et al., 2014, 2018).

Temperature compensation and temperature resetting of the clock is less well understood (Diernfellner et al., 2005). *frq* transcript levels remain the same at different temperatures but FRQ protein cycles at a higher mean level at high temperatures and due to alternative splicing a long form of FRQ is more prevalent (Liu et al., 1997; Diernfellner et al., 2005). Casein kinase 2 has been shown to have a role in temperature compensation and evidence suggests this is due to altered phosphorylation of FRQ at different temperatures (Mehra et al., 2009). *vvd* transcript and protein levels are also temperature-regulated, and play a role in temperature entrainment of the clock and temperature compensation of clock output pathways (Hunt et al., 2007). In addition, the existence of a temperature-compensated FLO that requires the components of the WCC has been described (de Paula et al., 2006; Hunt et al., 2012). An example of a temperature-compensated FLO-output is the clock-controlled gene *ccg*-*16*, which has been demonstrated to oscillate rhythmically in the absence of FRQ (de Paula et al., 2006).

Despite detailed knowledge of the *N. crassa* circadian clock, little is known about the existence of functional circadian clocks and their molecular basis in other fungi, although homologs of the *N. crassa* core circadian clock proteins, especially WC-1 and WC-2, have been found in other fungal species (Lewis and Feldman, 1996; Lombardi and Brody, 2005). It has been hypothesized that FRH and FWD-1 were present in the common ancestor of all fungi (Salichos and Rokas, 2010). WC-1 and WC-2 were probably gained in the common ancestor of Zygomycetes, Basidiomycetes, and Ascomycetes and subsequently lost in the Saccharomycetes. FRQ was likely gained in the ancestor of Ascomycetes and lost in Eurotiomycetes, remaining in Sordariomycetes, Leotiomycetes, and Dothideomycetes (Salichos and Rokas, 2010; Traeger and Nowrousian, 2015). Interestingly, FRQ is the least conserved of the clock proteins. While examples of functional *frq*-dependent circadian oscillators are present in the Leotiomycete *Botrytis cinerea* (Hevia et al., 2015) and in the Pezizomycete *Pyronema confluens* (Traeger and Nowrousian, 2015), other fungi, such as the Dothideomycete *Aureobasidium pullulans*, show no rhythmic *frq* expression, although they do display a circadian developmental rhythm (Franco et al., 2017). Circadian rhythms have also been demonstrated in species lacking a *frq* homolog, such as in *Aspergillus flavus* and *Aspergillus nidulans* (Greene et al., 2003). Furthermore, the yeast *Saccharomyces cerevisiae* was shown to exhibit circadian entrainment of metabolism despite the absence of a *frq* homolog (Eelderink-Chen et al., 2010), indicating a widespread presence of circadian oscillatory molecular processes across fungal species.

*Neurospora crassa* is phylogenetically related to many important plant pathogens including *V. dahliae*. *V. dahliae* is an asexual soil-borne Sordariomycete that causes wilt disease on more than 200 plant species worldwide, including high-value agricultural crops (Pegg and Brady, 2002). *Verticillium* sp. can persist in soil for long periods in the form of melanized resting bodies. In *V. dahliae*, these are clusters of thick-walled, septate and dark pigmented hyphal cells called microsclerotia (Isaac, 1949). Microsclerotia germinate upon the detection of plant root exudates, and colonize the vascular system. Once inside the vessels, conidia are produced and transported upward reaching extensive portions of the plant. Blockage of the transport system results in wilting of the plant and the subsequent formation of microsclerotia in the dead tissue, concluding the disease cycle.

The aim of this work was to determine whether a circadian clock is present in *V. dahliae*. As previously described (Heale and Isaac, 1965), under 24-h light/dark cycles *V. dahliae* displays concentric rings of conidia and microsclerotia. We report that this phenotype is directly driven by external cues rather than entrained. Comparative genetic studies between *N. crassa* and *V. dahliae* reveal that key clock components are conserved not only at the domain level, but down to individual phosphorylation sites. Similar sequences to the proximal and distal LRE motifs are present upstream of the VdFRQ ORF, although they are very widely spaced. However, qRT-PCR studies over a 48-h time-course revealed no rhythmic *Vdfrq* expression under constant conditions or in cycles of light/dark and high/low temperature. RNA-sequencing gene expression studies in the WT revealed large-scale changes in gene expression driven by changes from light to dark, but time-course RNA-sequencing revealed no strong signature of gene expression indicative of circadian rhythmicity. Our results show that while there is high conservation of clock components between *V. dahliae* and *N. crassa*, there is no strong evidence of a functional circadian clock in *V. dahliae*, at either the physiological or the molecular level.

## Materials and Methods

### Identification of *V. dahliae* Putative Clock-Genes

*Neurospora crassa* OR74A and *V. dahliae* JR2 genomes were downloaded from Ensembl Fungi (Kersey et al., 2016). Homologs were identified using BLASTp against the *V. dahliae* JR2 genome. Whole gene alignments between the query *N. crassa* gene and the *V. dahliae* JR2 hit were carried out in Geneious R10 using the MUSCLE algorithm. Next, *V. dahliae* JR2 hits were aligned to the genomes of five *V. dahliae* strains isolated and sequenced at NIAB EMR (12253, 12158, 12251, 12161, and 12008), available at DDBJ/EMBL/GenBank database using the following numbers: PRJNA344737 and PRJNA352681. Domains were identified within predicted proteins using InterProScan. Nuclear localization signals (NLS) were identified using cNLS Mapper. Identification of clock gene homologs in *Verticillium albo-atrum* (PD747), *Verticillium alfalfae* (PD683), *Verticillium nonalfalfae* (TAB2), *Verticillium longisporum* subgenome A (VLB2), *V. longisporum* subgenome D, *Verticillium nubilum* (PD621), *Verticillium tricorpus* (PD593), *Verticillium isaacii* (PD660), *Verticillium klebahnii* (PD401) and *Verticillium zaregamsianum* (PD739) was performed using BLASTp in collaboration with the Thomma Lab (Wageningen University).

### Orthology Analysis

The study involved 25 species, mostly plant pathogens, sampled from across the phylum Fungi. The respective genome (repeat-masked), CDS, and protein sequences were downloaded from Ensembl Fungi (Kersey et al., 2016). The orthology relationships between the genes in each genome were first established using the OrthoFinder ver. 1.0.7 (Emms and Kelly, 2015) and OrthoMCL ver. 2.0.9 (Li et al., 2003) pipelines. The genomes of 25 fungal species were searched for homologs of *frq* (NCBI ID: 3876095), *wc-1* (NCBI ID: 3875924), *wc-2* (NCBI ID: 3879968), *frh* (NCBI ID: 3872445), *fwd-1* (NCBI ID: 3872130), and *vvd* (NCBI ID: 3873728). The phylogenetic tree was built using a concatenated alignment of DI/D2 regions of large subunit (LSU) rDNA and ITS regions that were identified through BLAST and extracted from the genomes. The Tamura-Nei method (Tamura and Nei, 1993) was used to build trees, as implemented within Geneious R10.

### Promoter Motif Identification

2,000 bp upstream and downstream of each gene ORF were extracted. MEME suite ver. 4.11 was subsequently used to analyze the motif content of promoter regions (Bailey et al., 2009). For motif scanning purposes, the program FIMO (Grant et al., 2011) was used to deal with ungapped motifs (ACE motif in *ccg2* promoter), while gapped motif search for sequences showing similarity to the Clock-box element in *frequency* promoters was carried out with GLAM2SCAN (Frith et al., 2008). For *de novo* motif discovery, DREME (Bailey, 2011) and GLAM2 were used for ungapped and gapped motifs, respectively. Motif enrichment analysis was carried out with the AME program. A manual screen of consensus LRE motifs described in He and Liu (2005) and Smith et al. (2010) on the 2,000 bp upstream and 2,000 bp downstream regions of the *frq* orthologs across 13-species was performed using Geneious R10.

### *V. dahliae* Isolates and Growth Conditions

Strains of *V. dahliae* (Supplementary Table S1) were stored at −80°C as conidial suspensions preserved in 50% glycerol. Isolates were cultivated on petri dishes containing Prune Lactose Yeast Agar (PLYA) (Talboys, 1960) at 24°C in constant darkness. After a week, plates were flooded with 2 ml sterile water and gently rubbed using a plastic spreader to create a spore suspension.

### Plate Assays

Conidial suspensions were filtered using filter paper with 3–5 μm pore size and 1 μL of a 3 × 10^5^ spores/mL suspension was point-inoculated in the center of a PLYA plate and incubated for 14 days under the appropriate light and/or temperature conditions. For light entrainment experiments, after 14 days at 24°C in 12:12 LD, plates were marked at the end of the dark cycle before transfer to constant darkness (DD) for 7 days. For temperature entrainment assays, the cultures were incubated under 12 h at 20°C followed by 12 h at 28°C in the dark during 14 days prior to transfer to constant temperature. To assess the effect of entrainment by both light and temperature, plates were incubated in 12 h light at 28°C/12 h dark at 20°C, before transfer to constant darkness and temperature conditions. To examine nutritional compensation cultures were inoculated onto a high-nitrogen medium (PDA), low-nitrogen medium (Czapek DOX Agar), minimal medium (MM) (Hooykaas et al., 1979) and basal minimal medium (BMM) (Hu et al., 2013) and incubated in 12:12 LD cycles for 14 days. Strains were incubated in Panasonic MLR-352 or MIR-154 incubators equipped with broad spectrum fluorescent lamps FL40SSENW37. The light intensity was 75 μmol s^–1^ m^–2^ (approximately 5,600 lux). Colony size was measured using an electronic caliper. Each experiment was repeated at least three times and contained three replicas of each strain and treatment.

### Time-Course and Light Pulse Experiments

Rhythmic expression analysis by qRT-PCR in free-running conditions (constant darkness and constant temperature) was similar to the protocol described in Kramer (2007). A mycelial mat was formed by inoculating 1 × 10^8^ spores of *V. dahliae* into Petri dishes containing 20 ml of half strength PDB medium and incubated at 25°C under constant light for 96 h. Individual mycelial disks (1 cm diameter) were cut and inoculated in 100 ml Erlenmeyer flasks containing 25 ml of half strength PDB medium. A total of 36 flasks were used to cover 12 sequential time-points throughout two circadian cycles (4, 8, 12, 16, 20, 24, 28, 32, 36, 40, 44, and 48 h), each replicated three times. The flasks were incubated at 25°C in constant light for at least 24 h under agitation at 120 rpm. Cultures were staggered into constant darkness at different intervals, so that when harvested cultures were of similar ages. Cultures were harvested quickly (within 20 s) under red light. Red light alone or red light:dark cycles do not induce a developmental phenotype (data not shown). Disks were dried with filter paper, placed into 2 ml Eppendorf tubes, frozen in liquid nitrogen and stored at −80°C.

A similar methodology to that described in Nowrousian et al. (2003) was performed for light and temperature entrainment analysis. A total of 36 flasks were used to cover 12 sequential time-points throughout one LD cycle (L2, L4, L6, L8, L10, L11.5, D2, D4, D6, D8, D10, and D11.5). Similarly, a total of 36 flasks were used to cover 12 sequential time-points throughout one cycle of 12 h at 20°C (low temperature, LT)/12 h at 28°C (high temperature, HT) (L2, L4, L6, L8, L10, L11.5, H2, H4, H6, H8, H10, and H11.5).

For light pulse experiments, flasks containing half strength PDB medium were inoculated with *V. dahliae* or *N. crassa* mycelial disks and subsequently grown in constant light for 24 h at 25°C (120 rpm), after which they were transferred to constant darkness for 30 h prior to a 15-min exposure to white light (75 μmol s^–1^ m^–2^). After the light pulse, cultures were harvested under red light. Control flasks were not exposed to light pulses and were harvested under red safe-light conditions.

### RNA Extraction

Frozen mycelium of *N. crassa* and *V. dahliae* were ground with a mortar and pestle and total RNA extracted using the RNeasy Plant Mini Kit (Qiagen GmbH, Germany) according to the manufacturer’s instructions. The integrity of RNA samples was assessed using the Agilent 4200 TapeStation system (Agilent, Santa Clara, CA, United States).

### Gene Expression Analysis by Quantitative Real Time PCR (qRT-PCR)

cDNA from 1 μg of RNA was synthesized using the QuantiTect Reverse Transcription Kit (Qiagen, Germany) following the manufacturer’s instructions. qRT-PCR was performed with SYBR green (qPCRBIO SyGreen Mix Lo-Rox, PCR Biosystems, United Kingdom) and amplification followed using the CFX96^TM^ Real-Time PCR detection system (Bio-rad). Normalization was carried out against *V. dahliae* β-tubulin and Elongation factor 1-α transcripts. For *N. crassa*, β*-tubulin* and TATA binding box-encoding gene served as housekeeping genes. Primers are listed in Supplementary Table S2. PCR reactions were carried out in 10 μl containing 400 nM of each primer, 5 μl of 2x qPCRBIO SyGreen Mix and 2 μL of 1:4 diluted cDNA sample. The PCR conditions were 95°C 2 min; 39 cycles of 95°C 10 s, 60°C 10 s, 72°C 30 s and 1 cycle of 95°C 10 s, 65°C 5 s 95°C 5 s. Relative gene expression was calculated using the comparative cycle threshold (*C*_T_) method [2^–(ΔΔ^*^*C*^*^t)^] (Livak and Schmittgen, 2001). Expression values for free running time-course experiments were normalized to the cultures grown 4 h in the dark (D4). Expression values for time-course experiments under LD cycles were normalized to the cultures grown 2 h in the dark (D2), and cultures grown 2 h at 20°C (2L) were the reference samples for the temperature cycle/entrainment time-courses. The expression values for light pulsed cultures were normalized to cultures maintained in control conditions (DD).

Expression values derived from three biological replicates (unless otherwise stated) each containing three technical repeats, were analyzed using one-way Analysis of Variance (ANOVA). The residuals were tested for normality and if required, data were log-transformed. Statistical analyses were carried out using R Studio software. Statistical analysis of rhythmicity of time-course experiments was achieved with JTK-CYCLE (Hughes et al., 2010) in the R software (version 3.3.0) using the 2^–(ΔΔ^*^*C*^*^t)^ normalized data of all the replicates. The analysis was performed as described in the JTK-CYCLE manual, looking for period lengths of 24 h.

### RNA-Seq

For the time-course RNA-seq experiment in free-running conditions, samples of *V. dahliae* 12253, 12008 and Δ*Vdfrq_12253* were harvested under red light after 6, 12, 18, and 24 h in the dark. Concurrently, for light versus dark analysis, cultures were incubated in constant darkness and then were transferred to white-light and harvested after 6 h. The experiment contained three biological replicates of each strain and condition.

Total RNA was extracted as described above. For transcriptome sequencing, samples with a minimum of 1 μg of RNA (100 ng/μl), ≥6.8 RIN and 260/280 nm values > 1.8 were sent to Novogene Technology Co. Ltd. (Wan Chai, Hong Kong). Sequencing was performed on Illumina HiSeq P150.

Quality control of the RNA-seq reads was carried out by fastQC and adapters were trimmed using fastq-MCF^1^. STAR software (Dobin et al., 2013) was used to align RNA-seq reads to the reference *V. dahliae* JR2 genome (Ensembl Fungi). Mapped read counts were calculated using the program featureCounts (Liao et al., 2013). The analysis of expression of all predicted genes was performed with the DESeq2 package in R. A grouping command was used to assess differentially expressed genes (DEG) between conditions (L or D) and strains (Vd12253, Vd12008, and Δ*Vdfrq_12253*). The false discovery rate (FDR) cut-off was set to 0.05. Genes were considered to be significantly differentially expressed when *p*-value < 0.05 and presented more than 1-log2 fold change (LFC) in transcript level.

Principal component analysis (PCA) plot and samples distances plot used rlog-transformed read counts, and were carried out using R. Gene ontology (GO) terms were retrieved from GO.db in R. Gene enrichment analysis for GO terms was performed using topGO in R with Fisher’s exact test to retrieve significantly enriched processes of the DEG. The analysis of secondary metabolite biosynthetic gene clusters in *V. dahliae* was performed using antiSMASH (Blin et al., 2016).

For rhythmic expression analysis, the raw counts of the time-course samples (6, 12, 18, and 24 h) were normalized to fragments per kilobase of transcript per million mapped fragments (FPKM). Normalized expression values of gene orthologs of the central clock-oscillator, clock-controlled genes, and photoreceptors genes over the four time-points were analyzed using a two-way Analysis of Variance (ANOVA). The residuals were tested for normality and if required, data were log-transformed. Statistical analyses were carried out using R Studio software.

### Construction of *V. dahliae frq* Replacement Cassette

The 5′ and 3′ flanking regions of the *Vdfrq l*ocus were amplified from genomic DNA using primer pairs HRFrq1-F/R and HRFrq2-F/R, respectively (Supplementary Table S2). Core USER Bricks and the *hygromycin* resistance cassette were amplified from pRF-HU2 plasmid and the vector bricks were assembled through USER reaction and transformed into *Escherichia coli* DH5α competent cells (Sørensen et al., 2014). The resulting vector junctions were amplified using B1.B2-F/R, B1.B2.F1.H-F/R, and H.F2.B1-R to confirm the correct assembly.

### *Agrobacterium tumefaciens* Transformation

*Agrobacterium tumefaciens* EHA105 was transformed with the *Vdfrq* replacement vector as described in Höfgen and Willmitzer (1988). Putative transformants were checked by PCR using primers B1.B2-F/R, B1.B2.F1.H-F/R and H.F2.B1-R (Supplementary Table S2).

### Agrobacterium-Mediated Transformation of *V. dahliae*

*Agrobacterium tumefaciens* EHA105 containing the pEcFrq-D1 binary vector was grown at 28°C for 48 h in MM containing 50 μg ml^–1^ of rifampicin and 50 μg ml^–1^ of kanamycin on a rotary shaker (200 rpm). At an optical density of OD_600_ = 0.5, bacterial cells were diluted to OD_600_ = 0.15 with IM (Bundock et al., 1995) containing 200 μM acetosyringone (AS). Cells were grown for an additional period of 12–15 h before being mixed with an equal volume of a spore suspension (1 × 10^6^ spores ml^–1^) of the required strain of *V. dahliae*, previously grown for 3 days in flasks containing PDB liquid media in a rotary shaker 180 rpm in the dark. From this mixture aliquots of 200 μl were plated on a cellophane membrane placed on an IM agar plate. After incubation at 25°C for 48 h the membrane was transferred onto a PDA plate containing 200 μg/ml tricarcillin and 50 μg/ml hygromycin B and incubated at 24°C for a further 5–7 days. Discrete colonies were selected and grown for 24 h in PDB liquid cultures containing 50 μg/ml hygromycin B. Single-spore colonies were obtained by plating 100 μL of the culture on PDA with 50 μg/ml hygromycin B. PCR was carried out on selected single-spore mutants to confirm insertion of hygromycin using the primers TestHygr-F/R and FrqUS_Hygr-F/R, and lack of product from deleted gene using the primers TestFrq-F/R (Supplementary Figure S1). The PCR conditions using Taq 5x Master Mix (New England Biolab) were 95°C 30 s; 35 cycles of 95°C 20 s, 60°C 20 s, 68°C 2 min, and 68°C for 5 min.

### *Arabidopsis thaliana* and Strawberry *in vitro* Propagation

For pathogenicity tests, seeds of *Arabidopsis thaliana* ecotype Columbia (Col), were surface-sterilized by sequential immersion in 70% ethanol for 1 min and 10% (v/v) bleach containing 0.1% (v/v) Tween-20 for 5 min with gentle shaking. Seeds were then washed with autoclaved distilled water for 5 min five times, re-suspended in sterile 0.1% agarose, and subsequently stratified at 4°C over a period of 2–3 days in darkness (Zhang et al., 2006). Five to six seeds were pipetted into 120 × 120 mm square petri dishes (Thermo Fisher Scientific) containing half-strength Murashige and Skoog salts (MS), pH 5.7; 0.8% (wt/vol) Phytagel (Sigma-Aldrich); 1% sucrose; and grown at 22°C under 16:8 LD cycles in a Panasonic MLR-352 incubator for 3–4 weeks.

Strawberry (*Fragaria* x *ananassa*) cultivar Hapil plantlets were preserved in sterile jars containing SMT proliferation medium [MS salts 4.41 g, 177.5 μM Benzylaminopurine (BAP), 254 μM Gibberellic acid (GA), 255 μM Indole butyric acid (IBA), sucrose 30 g, agar (Oxoid 3) 7.5 g, adjust pH = 5.6]. The optimal growth conditions were 24°C in the light and 19°C in the dark, in a photoperiod of 16 h light and 8 h dark. For long-term *in vitro* material, plantlets were transferred to fresh SMT medium in sterile conditions every 6 weeks. For root induction, SMR rooting medium [MS salts 4.41 g, 254 μM GA, 255 μM IBA, sucrose 30 g, agar (Oxoid 3) 7.5 g, adjust pH = 5.6] was used. Four weeks prior to a pathogenicity test, roots were carefully removed from the media just below the crown and the tips were moved to square petri dishes containing ATS medium as specified in Williamson et al. (2001), where subsequent roots were produced.

### *Arabidopsis thaliana* and Strawberry Root Inoculation Assay

*Verticillium dahliae* conidial suspensions were harvested from 7-day old liquid cultures and diluted to 1 × 10^6^ spores/ml with PDB. Three to 4-week-old *Arabidopsis* seedlings were moved under aseptic conditions to new plates containing half-strength MS salts pH 5.7; 0.8% (wt/vol) Phytagel (Sigma-Aldrich) without sucrose, and root tips were trimmed using sterilized scissors. Each plate contained five *Arabidopsis* seedlings that were inoculated with 200 μL of the spore suspension spread onto the roots. In mock plates seedlings were inoculated with 200 μL of autoclaved distilled water. Plates were subjected to a light-dark cycle of 12:12 h and constant temperature of 24°C for the duration of the experiment.

For the strawberry inoculation, plants were removed from the plates and the roots were carefully cleaned free of agar with a sterile blade. Before the inoculation, 1 cm of roots was trimmed from the bottom with sterile scissors. Plants were inoculated by dipping the root systems into a flask containing 1 × 10^6^ spores/ml suspension (6 plants/100 ml inoculum) for 5 min. A fresh spore suspension was used for each batch of 6 plants. Plants were then placed to new ATS medium plates sealed with tape. Uninfected plants were inoculated with sterile distilled water following the same procedure as stated before. Plates were subjected to a light-dark cycle of 12:12 h and temperature of 24°C in a Panasonic MLR-352 incubator.

### Disease Assessment

*Arabidopsis* and strawberry cv. Hapil seedlings were photographed and scored for disease symptoms using a scale with nine categories modified from Eynck et al. (2007); Score 1: Healthy plant, 2: Slight symptoms on older leaves (yellowing), 3: 1–2 outer leaves affected, 4: >2 leaves affected, 5: 50% leaves affected, 6: >50% leaves affected, 7: 75% leaves affected, 8: >75% leaves affected, 9: Plant dead.

## Results

### Distribution of Core Clock Gene Homologs in *Verticillium* Species and Other Sordariomycete, Dothideomycetes, and Leotiomycetes of Economic Importance

**FIGURE 1:**
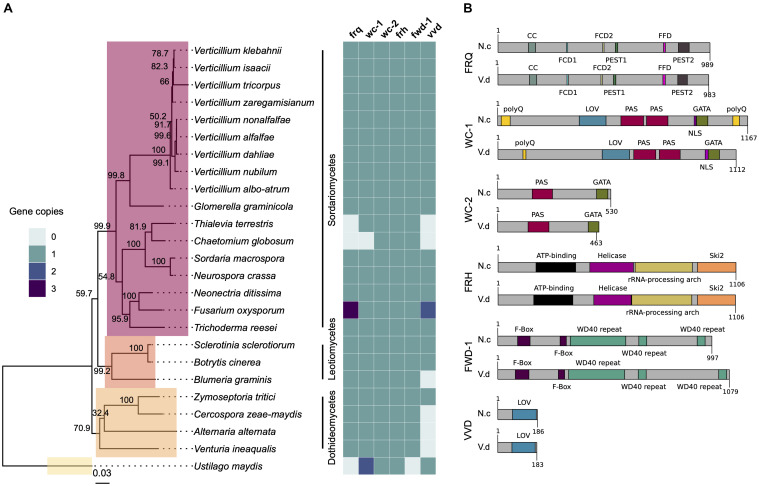
The circadian clock components are conserved across fungal species. **(A)** *Neurospora crassa* clock gene homologs across 25 fungal genomes. The presence, absence and copy number is shown for core clock genes (*frq*, *wc-1*, *wc-2*, *frh*, *fwd-1*, and *vvd*) on the heatmap on the right. The phylogenetic tree was constructed based on the concatenated D1/D2 and ITS sequence alignment using the Neighbor-Joining method based on the Tamura-Nei model with Geneious R10 software. Bootstrap support values from 1,000 replicates are shown at the nodes. The fungal classes are shown in color boxes on the tree. The Basidiomycete *Ustilago maydis* was used as outgroup species. **(B)** Protein domain structure of core clock proteins in *N. crassa* and *V. dahliae* based on InterProScan database. FRQ, WC-1, WC-2, FRH, FWD-1, and VVD are highly conserved at the domain level. Protein length (amino acids) is displayed at the end of each protein sequence. CC, coiled-coil; FCD, FRQ-CK1a interaction domains; PEST, proline (P), aspartic and glutamic acid (E), serine (S), threonine (T)-rich protein turnover; polyQ, poly-glutamine stretch domain; LOV, light-oxygen-voltage sensing; PAS, Per-Arnt-Sim, protein binding domain; GATA, Zinc finger, GATA-type; DNA, binding domain; helicase, ATPase activity; F-box, protein–protein interaction motif; WD40 repeat, interacting domain.

The genomes of 25 Sordariomycete, Dothideomycete, and Leotiomycete species were searched for homologs of *frq*, *wc-1*, *wc-2*, *frh*, *fwd-1*, and *vvd*. The predicted proteins from these genomes were clustered and proteins in the same orthogroup cluster as the *N. crassa* genes were considered orthologous genes. Orthologs of all six clock oscillator genes are present in most species of Sordariomycetes tested in this study, including important plant-pathogenic fungi such as *Glomerella graminicola* and *Neonectria ditissima* (Figure 1A). Homologs of clock genes are present in all *Verticillium* species tested: *V. albo-atrum*, *V. alfalfae*, *V. nonalfalfae*, *V. dahliae*, *V. nubilum*, *V*, *tricorpus*, *V*. *isaacii*, *V. klebahnii*, and *V. zaregamsianum* (pers. comm. B. Thomma). *Fusarium oxysporum* has three copies of *frq* and *vvd*. Conversely, members of the chaetomium family appear to have lost *frq*, *wc-1*, and *vvd* homologs. In contrast to *Sclerotinia sclerotiorum* and *Botrytis cinerea*, the Leotiomycete *Blumeria graminis* lacks a *vvd* homolog, but contains copies of the other clock components. *vvd* is absent in the Dothideomycetes *Cercospora zeae-maydis*, *Alternaria alternata*, and *Venturia inaequalis*, but present in the wheat pathogen *Zymoseptoria tritici* that contains homologs of all the clock genes (Figure 1A). The genome of the Ustilaginomycetes *Ustilago maydis* does not harbor an ortholog of *frq*, in agreement with the findings in (Salichos and Rokas, 2010).

Alignments of core clock proteins between *N. crassa* and *V. dahliae* revealed sequence identities greater than 43% and query coverages greater than 39%. Consistent with previous reports (Traeger and Nowrousian, 2015), *V. dahliae* FRQ is the least conserved (43.82% identity), and FRH is the best conserved (68.69% identity) of the core clock proteins. Clock proteins are strongly conserved at the domain level (Figure 1B). Although the FRQ protein alignment created between *N. crassa* and *V. dahliae* sequences shows moderate conservation overall, a number of important regions are highly conserved (Supplementary Figure S2A). In *N. crassa* PEST1 and PEST2 are involved in determination of period length and cytoplasmic accumulation of the WCC (Görl et al., 2001; Schafmeier et al., 2006). Whilst a high degree of variation within VPEST2 exists, VPEST1 is better conserved. The formation of FRQ homodimers, essential for clock function, is mediated through its coiled-coil domain (Cheng et al., 2001). Between *N. crassa* and *V. dahliae* this domain shares 69.69% identity. FRQ-CK1a interaction domains, FCD1 and FCD2 (He et al., 2006; Querfurth et al., 2011) share 87.5% identity between *N. crassa* and *V. dahliae.* In contrast, low sequence conservation of the FRQ-FRH interaction domain (FFD) (Guo et al., 2010) is observed between *N. crassa* and *V. dahliae*, with 4 of 10 sites conserved. *V. dahliae* FRQ protein contains two predicted nuclear localization signal (NLS) sequences, one almost identical to the *N. crassa* NLS (DLLKRDKLFEIKVHGLPKPKKRELE). The NLS present in *N. crassa* FRQ is required for its entrance into the nucleus and down-regulation of *frq* transcription (Luo et al., 1998). Furthermore, of the 73 *in vivo* and *in vitro* identified phosphorylation sites in *N. crassa* FRQ (Tang et al., 2009), 40 sites are conserved in *V. dahliae* FRQ. Of note Ser 513, a determinant of period length (Liu et al., 2000) and FRQ degradation, is present in *Vd*FRQ.

Interestingly, alignment of *frq* homologs from six *V. dahliae* strains (JR2, 12253, 12251, 12008, 12161, and 12158) revealed 15 single nucleotide polymorphisms (SNPs). The same 15 SNP are found in strains 12161 and 12158 belonging to vegetative compatibility (VC) subclade II-2 and are absent in strains JR2, 12253, 12251, and 12008 belonging to VC subclade II-1 (Supplementary Figure S2B). Consequently, VdFRQ protein alignment indicates four amino acid substitutions however, none of these substitutions are in the known functional domains.

In *N. crassa*, WC-1 and WC-2 interact through their PAS domains to form a heterodimeric complex (WCC complex). The WCC is essential for light-induced gene expression of *frq* and other light-regulated genes, and is required for the generation of circadian rhythms (Cheng et al., 2001, 2002). *V. dahliae* WC-1 and WC-2 contain all the domains required for their interaction, light perception and transcription factor (TF) activity (Figure 1B). *Vd*WC-1 and *Vd*WC-2 share 45.22 and 51.12% sequence identity with the *N. crassa* homologs, respectively. Nevertheless, *Vd*WC-1 is highly conserved (79.0–94.34% identity) in the known protein domains. *Vd*WC-1 contains a conserved Light-oxygen-voltage (LOV) domain. This domain is related to the Period-ARNT-Single-minded (PAS) domain family, and is distinguished by binding a flavin cofactor and the presence of a GXNCRFLQ motif (Taylor and Zhulin, 1999). Furthermore, VdWC-1 contains two highly conserved PAS domains required for protein interaction, and a GATA-type zinc finger DNA binding domain commonly present in GATA-type transcription factors (Supplementary Figure S3). A region of basic amino acids (LLSNKKKRKRRKGVG) required for rhythmicity and circadian expression of the Neurospora circadian clock gene *frequency* is also present (Wang et al., 2016). Despite the high level of conservation of most WC-1 domains between *N. crassa* and *V. dahliae*, the conservation is poor in the first 480 aa. In *N. crassa*, this region contains a poly-glutamine (Poly-Q) region expansion aa 16–61 that has previously been observed in transcription factors and is reported to play a role in transcription activation and to affect period length (Ballario et al., 1996; Michael et al., 2007). Other reports suggest that neither the N nor C-terminal polyQ stretches are required for activation of *frq* transcription, and it is an adjacent region spanning aa 100–200 in WC-1 that is necessary for clock control of *frq* expression in the dark but not for light responsiveness of *frq* (Wang et al., 2014). In *V. dahliae*, however, the polyQ stretch is not continuous and conservation is minimal in the aa 100–200 region. Furthermore, *V. dahliae* WC-1 lacks the C-terminal (Poly-Q) region present at the aa 1,091–1,133 in *N. crassa*.

*Vd*WC-2 contains a single PAS domain, a GATA-type zinc finger transcription factor domain with 65.71 and 79.24% sites conserved. A putative NLS (RGRKRKRQW) sequence identified in *V. dahliae*, lacks a homologous sequence in *N. crassa* (Supplementary Figure S4).

### *V. dahliae* Conidiation and Microsclerotia Production Are Light-Regulated but Not Under Control of a Circadian Clock

**FIGURE 2:**
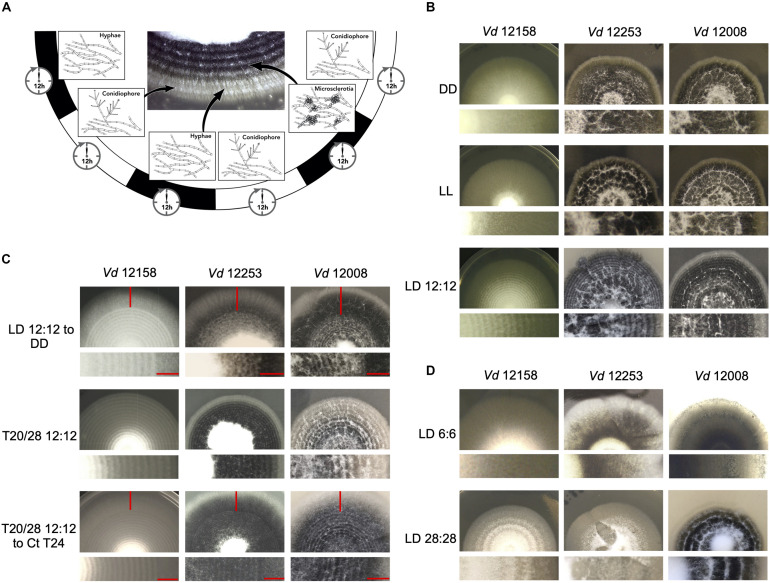
*Verticillium dahliae* morphological rhythms do not free-run. **(A)** Development of *V. dahliae* in 12 h light: 12 h dark cycles (LD 12:12). After a light period, conidiophores (white bands) are developed above the hyphae, but never on the leading edge. Microsclerotia start to develop 48 h after inoculation, and are produced during the dark phase of the cycle. This leads to concentric zones of conidia and microsclerotia. **(B–D)** Colonies of *V. dahliae* strains on PLYA plates, bottom panels magnified sections of plate colonies. **(B)** *V. dahliae* morphological rhythm under different light regimes. *V. dahliae* hyaline isolate 12158 and the microsclerotia producer isolates 12253 and 12008 were point-inoculated on PLYA plates and grown in constant darkness (DD); constant white light (LL); cycles of 12 h white-light/12 h dark (LD 12:12). All plates were incubated at 24°C. Experiments were performed five times. **(C)** Strains 12158, 12253, and 12008 point-inoculated on PLYA plates and grown under light cycles of 12 h white-light:12 h dark at 24°C or under temperature cycles of 12 h 20°C: 12 h 28°C (T20/28 12:12) for 14 days, were transferred to constant darkness (LD12:12-DD) or constant temperature at 24°C (T20/28 12:12 – Ct24) for 7 days. Red horizontal lines indicate the period of growth under constant conditions. **(D)** *V. dahliae* exposed to short and long T cycles. *V. dahliae* isolates 12158, 12253, and 12008 were point inoculated onto PYLA plates and incubated for 14 days at 24°C under T cycles of LD 6:6 and LD 28: 28. *V. dahliae* produces very faint rings when grown under 6:6 LD cycles.

*Verticillium dahliae* produces visible concentric rings of conidia and microsclerotia when cultured in cyclic environments, such as light/dark (LD) and high/low temperature cycles (Heale and Isaac, 1965). Under 24 h LD cycles at constant temperature, hyphal growth occurs under both light and darkness, however, conidiation from hyphae is induced after a period of light (Figures 2A,B). After a minimum of 48 h of growth microsclerotia form but in LD cycles they are mainly produced in the dark (Heale and Isaac, 1965). Nevertheless, in either constant darkness (DD) or constant light (LL) conditions *V. dahliae* cultures contain conidia and microsclerotia but they are not produced in rings (Figure 2B). To test if development is controlled by a circadian clock we investigated whether the morphological rhythms found in this pathogen are endogenous and can be entrained. We found that *V. dahliae* cultures transferred to DD after an entrainment period of 12:12 LD for 14 days at 22°C cease to produce rings of conidia and microsclerotia. In 12:12 temperature cycles of 20 and 28°C in DD or LL the banding pattern is synchronized to the temperature oscillations, and ceases when cultures are transferred to constant temperature (Figure 2C). Thus, the developmental rhythms are induced by light and temperature cycles, rather than being self-sustainable.

Circadian clocks are able to entrain to cyclical cues in the environment and in all organisms studied to-date, light experienced late in the subjective day causes phase delays of clock time whilst light experienced late in the subjective night causes phase advances. This affects rhythms that are clock-controlled such that in changing day-lengths clock-controlled outputs occur at the correct time of day (Pittendrigh, 1960). This ability to reset also allows circadian clocks to entrain to environmental cycles shorter and longer than 24 h (T cycles). A phenomenon known as frequency demultiplication emerges when organisms are exposed to very short or long periods (e.g., 6:6 or 24:24 LD or temperature cycles). That is, they display a 24-h rhythm as if the oscillation is entrained to a 12:12 cycle (Pittendrigh, 1960). In contrast, if a rhythm is simply a direct response to external cues, it assumes the periodicity of the driving light or temperature cycle (Pittendrigh, 1960). In *V. dahliae*, cultures grown under 6:6 LD cycles show narrow conidial and microsclerotia bands, whereas cultures under 28:28 LD form widely spaced bands (Figure 2D). Thus, the developmental rhythm shows no evidence of frequency demultiplication. Instead, conidial and microsclerotia production follows each light/dark and temperature transition.

To establish whether the lack of free-running morphological rhythms was representative of a wider set of *V. dahliae* isolates, 12 different isolates were tested under light/dark and temperature 20°C/28°C cycles (Supplementary Figure S5). Although all the isolates respond to both light and temperature cycles, in the absence of cyclic light or temperature there is no evidence of free-running rhythms. Data collected from one isolate of a species is not necessarily representative of a species (Fuller et al., 2016), yet alone different species. For this reason, we characterized the photobiology and tested for the presence of circadian rhythms in three additional species in the *Verticillium* genus: *V. albo-atrum*, *V. nubilum*, and *V. tricorpus*. When cultures were treated in an alternating 12 h light/12 h dark photocycle, or 20°C/28°C cycles in constant darkness, concentric rings of conidia and resting structures were formed. However, as in *V. dahliae*, there is no apparent circadian rhythm of development on transfer to constant conditions in any of the Verticillium species tested in this study (Supplementary Figure S6).

### *V. dahliae* Lacks Rhythmic *Vdfrq* Gene Expression in LD and DD

**FIGURE 3:**
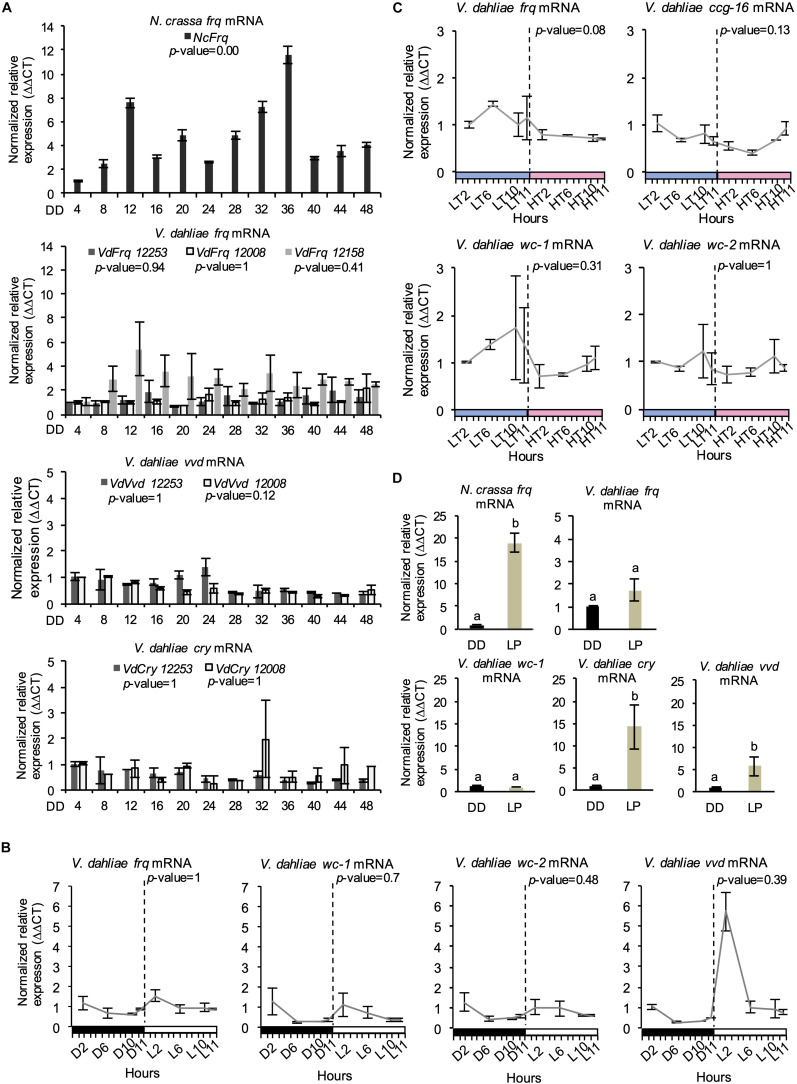
Clock-gene expression profile in *V. dahliae*. **(A)** Time-course expression of *N. crassa frq* and *V. dahliae frq*, *vvd* and *cry* under free-running conditions (DD, 24°C) in different isolates (12253, 12008, and 12158). *Vdfrq*, *Vdvvd* and *Vdcry* transcript levels were assessed by qRT-PCR every 4 h over a period of 48 h in constant darkness (DD). *N. crassa* 30-7 *bd* (first graph) was utilized as an experimental control. The β-tubulin and Elongation factor 1-α genes were the *V. dahliae* housekeeping genes. The *N. crassa* housekeeping genes were the β-tubulin and TATA binding box-encoding genes. Transcript levels are normalized to ddCt of DD4 samples in each strain (control = 1). Data is presented as mean (±SEM) from three independent biological replicates. **(B)** Gene expression analysis of four *V. dahliae* putative clock genes (*Vdfrq*, *Vdwc-1*, *Vdwc-2*, and *Vdcry*). Cultures were exposed to 12 h:12 h LD cycles and *V. dahliae* 12253 tissue was harvested at different time-points in the dark (D) or light (L) over 24-h. The dashed vertical lines represent the transition from dark to light conditions. Black-white bars indicate the dark-light conditions, respectively. β-tubulin and Elongation factor 1-α genes were used as housekeeping genes against which clock gene signals were normalized. Transcript levels are normalized to ddCt of D2 conditions for each gene (control = 1). Data is presented as mean (±SEM) from three independent biological replicates. **(C)** Gene expression analysis of putative clock and clock-controlled genes under (*Vdfrq*, *Vdwc-1*, *Vdwc-2*, and *Vdccg-16*) under cyclic temperature conditions. Cultures were entrained to 12 h/12 h 20°C/28°C temperature cycles, and *V. dahliae* 12253 mycelial tissues were harvested at different time-points at low (20°C, LT) or high (28°C, HT) temperatures over a 24-h period. The dashed vertical lines represent the transition from L to H conditions. Blue–pink bars indicate the low–high temperature conditions, respectively. β-tubulin and Elongation factor 1-α genes were used as housekeeping genes. Transcript levels are normalized to ddCt of L2 conditions for each gene (control = 1). Data is presented as mean (±SEM) from three independent biological replicates. **(D)** qRTPCR expression analysis of *Vdfrq*, *Vdwc-1*, *Vdcry*, and *Vdvvd* in the dark and in response to a 2-min light pulse. After 36 h in constant dark at 25°C, half of the cultures were given a 15-min light pulse after which they were harvested. Data are presented as means (± SEM) with different letters indicating statistically significant differences (*p*-value < 0.05) between samples.

A characteristic property of circadian rhythms is their persistence in constant conditions, and rhythmic expression of *frq* in continuous darkness has been widely used as a marker of circadian time (Bell-Pedersen et al., 1996). In *V. dahliae* isolates 12253, 12008, and 12158, *Vdfrq* mRNA levels under free-running conditions are arrhythmic (*p*-value = 0.94, *p*-value = 1, and *p*-value = 0.41, respectively). In contrast, exposed to the same conditions, robust oscillation of *N. crassa frq* is seen (Figure 3A) and, in agreement with previous studies (Aronson et al., 1994), *frq* expression peaked at 12 and 36 h after transfer to DD (subjective morning). Another feature of circadian rhythms is that they anticipate cyclic changes in the environment. Therefore, we assessed the expression of several clock genes (*frq*, *wc-1*, *wc-2*, and *vvd*) in a 12:12 LD cycle, with high time-point resolution before and after “lights on” (Figure 3B). *Vdfrq* transcript levels under DD seemed to anticipate the transition to light as the expression slightly increased from D10 to D11.5, albeit with less than a 1 log_2_ fold change. However, *Vdfrq* transcript levels do not exhibit significant rhythmic oscillation (*p*-value = 1) and drop after the first time-point in the light. There is a slight increase in the expression of *Vdwc-1* and *Vdwc-2* after 2 h in the light, whereas *Vdvvd* is highly expressed after the dark to light transition, and drops to basal levels at later time-points possibly due to photoadaptation (Schwerdtfeger and Linden, 2003) (Figure 3B). In conclusion, there is no strong evidence for anticipatory behavior that could indicate light entrainment of a *V. dahliae* circadian clock, nor is there significant rhythmic gene expression in LD. Similarly, there is no significant difference in the expression of *Vdfrq*, *Vdwc-1*, *Vdwc-2*, or *Vdccg-16* before or after the transition between a high temperature (HT) period of 28°C or a low temperature (LT) period of 20°C (Figure 3C).

### Promoter Clock Box and LRE Motif Identification

In *N. crassa* the expression of *frq* is rapidly induced by pulses of light, mediated by the WCC, enabling light entrainment of the clock and synchronization to external conditions (Crosthwaite et al., 1995, 1997). The presence of key clock genes in Verticillium but absence of observable clock-driven developmental rhythms prompted us to look at the promoter regions of *Vdfrq* and *Vdqrf*. For comparison, we included in our search the *frq* and *qrf* promoters of a subset of fungal species known to have a functional circadian clock, such as Botrytis (Hevia et al., 2015) and Magnaporthe (Deng et al., 2015). Two experimentally verified WCC binding motifs in the promoters of Neurospora clock-related genes containing two imperfect GATN repeats (Froehlich et al., 2002, 2003) were identified in our *in silico* analysis: the proximal and distal light regulatory element (LRE) motifs containing the sequence 5′GATNC–CGATN3′, where *N* is the same in both repeats (He and Liu, 2005), and 5′GATCGA3′ (Smith et al., 2010).

Putative proximal or distal motifs containing the sequence 5′GATNC–CGATN3′ are found in the *frq* promoter region (2,000 bp upstream the ORF) of all fungal species analyzed in this study (Supplementary Figure S7). However, in most species, including *V. dahlia*e, the distance between the two LRE motifs is more than 70 bp, very far from the 3 and 11 nucleotide gap found in the functionally verified proximal and distal *N. crassa frq* promoter motifs (He and Liu, 2005). The clock-containing organism *B. cinerea* presents a putative proximal motif with a 5-nucleotide gap and *N. ditissima* shows two 4 nucleotide-gapped putative motifs within the first 400 nucleotides upstream the ORF. Interestingly, *V. tricorpus*, *V. zaregamsianum*, and *V. albo-atrum* also present putative motifs with a short gap of maximum 14 nucleotides but they strongly differ in the position within the promoter. On the other hand, *Magnaporthe poae* presents a putative motif with a gap length of 55 nucleotides.

A perfect match to the proposed WCC binding motif, 5′GATCGA3 ′, described by Smith et al. (2010) was found several times in the majority of the promoters. In *N. crassa*, the motif is found in the antisense sequence and close to the gene start site, which is also observed in *M. poae*, *V. nonalfalfae*, *V. alfalfae*, *V. dahliae*, and *V. albo-atrum*. At the terminator level, *N. crassa* presents a WCC binding motif that induces the transcription of an antisense *frq* sequence known as *qrf* (Xue et al., 2014). In our study, only *N. ditissima* and *V. isaaci* show a gapped motif in the antisense sequence, although they are considerably distant from the stop codon. Overall, our analysis shows that the conservation in number, position and sequence of promoter motifs does not necessarily correlate with the presence of an active circadian clock.

### Light Does Not Activate the Transcription of *Vdfrq* but Regulates Expression of Photoreceptors *Vdcry* and *Vdvvd*

We then tested the ability of light to induce expression of *frq* in *V. dahliae*. The mycelium of *V. dahliae* 12253 strain was grown in shaking liquid media in constant darkness (DD). Then, the cultures were either kept in the dark (dark control) or transferred to white light for 15 min after which RNA levels were assessed by qRT-PCR (see methods). Our results reveal that *V. dahliae frq* expression is not induced by pulses of light (Figure 3D), whereas, as expected, *N. crassa frq* transcript increases 10-fold. Furthermore, in contrast to *N. crassa* (Ballario et al., 1996), the expression of *Vdwc-1* also lacks induction by light (Figure 3D). In contrast, orthologs of the light-inducible *N. crassa* genes *vvd* and *cry* are both up-regulated. *Vdcry* mRNA increases 14-fold and *Vdvvd* mRNA 5-fold in response to light (Figure 3D).

### No Indication From RNA-Seq Data That Putative *V. dahliae* Clock-Associated Transcripts Are Clock-Controlled

To look at a larger number of clock-associated transcripts for patterns of gene expression indicative of circadian rhythmicity we analyzed 24 h RNA-Seq data. RNAseq was performed with 12253 and 12008 because of the three *V. dahliae* strains used in this study, 12253 and 12008 are the most pathogenic in strawberry. If a circadian clock influences infection then differences in gene expression, timing and extent of infection in these strains would be the most informative. Following transfer of cultures from light to dark RNA-Seq analysis was carried out in three biological replicates harvested at DD6, DD12, DD18 and DD24 in wild-type (12008 and 12253) strains.

**FIGURE 4:**
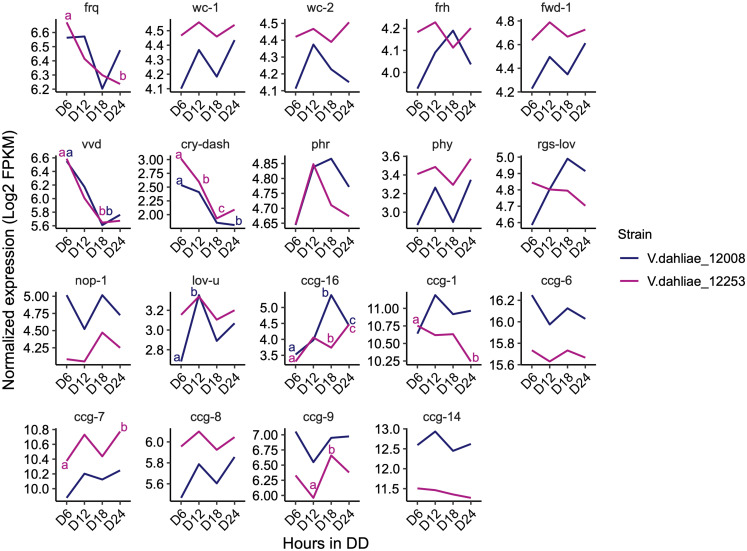
Expression pattern of *N. crassa* homologs of clock oscillator and clock-controlled genes (ccg) in both *V. dahliae* 12253 and 12008 over 24-h in the dark. Normalized expression (Log_2_ FPKM) is shown. The scales are different for every gene. A two-way ANOVA was performed. Data are presented as means with different letters indicating statistical differences (*p*-value < 0.05). Only significant statistical differences are marked.

The expression of most genes encoding orthologs of the central clock-oscillator (*frq*, *wc-1*, *wc-2*, *frh*, and *fwd-1*), clock-controlled genes (*ccg-1*, *ccg-6*, *ccg-7*, *ccg-8*, *ccg-9*, and *ccg-14*), and photoreceptors (*phr*, *phy*, *rgs-lov*, *nop-1*, and *lov-u*) are not significantly different over the 24-h time course. Transcripts of *frq*, *lov-u*, *ccg-1*, *ccg-7*, and *ccg-9* do show significant differences in one of the two strains but for most of these transcripts the difference is considerably less than 1 log_2_-fold change. Differences in transcript levels of *vvd*, *cry-dash*, and *ccg-16* are significant in both strains. *Vvd* and *cry-dash* decrease by approximately 1 log_2_-fold change from 6 to 18 h in darkness reflecting a decrease to dark-adapted levels (Figure 4). In Neurospora *vvd* transcript levels also decrease rapidly on transfer from light to dark but thereafter peak at DD12 and DD36 (Heintzen et al., 2001). However, the data in Figure 3 show that *V. dahliae vvd* and *cry* transcript levels do not change rhythmically over the course of 48 h in constant dark. *ccg-16* transcripts show the largest difference, in 12008 levels increase approximately 1.5 log_2_-fold after 18 h in the dark but in a 48-h time course are not rhythmic (data not shown). Thus, whilst extremely low amplitude rhythms may be present there is no indication from our 24-h RNA-Seq and qRT-PCR data that the expression of putative *V. dahlia*e clock-associated or photoreceptor transcripts are clock-controlled.

### *V. dahliae frq* Affects Fungal Growth

**FIGURE 5:**
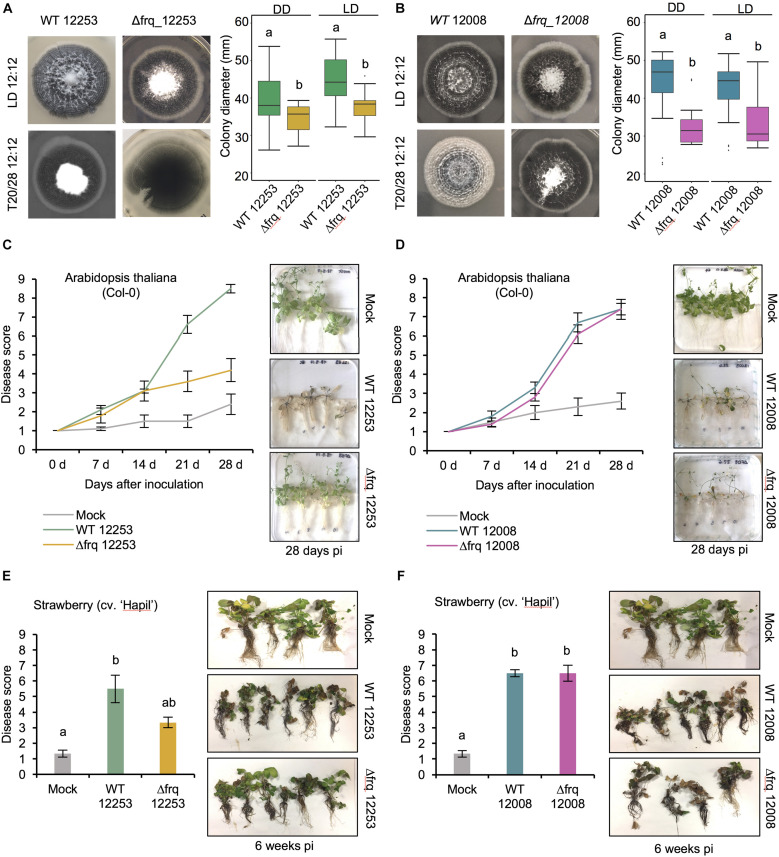
The effect of *Vdfrq* deletion mutants on fungal growth and pathogenicity. **(A)** Phenotype of *V. dahliae frq* deletion in 12253 and **(B)** 12008 background. PYLA plates point inoculated with WT 12253, Δ*frq_12253*, WT 12008 and Δ*frq_12008* and incubated in LD 12:12 and temperature (20/28) 12:12 cycles for 14 days. Boxplot graphs depict WT 12253, Δ*frq_12253*, WT 12008 and Δ*frq_12008* colony diameters in DD and LD, *n* = 6. Letters indicate statistical differences (*p*-value < 0.05), Tukey’s HSD test. **(C)** Disease score of *A. thaliana in vitro* seedlings inoculated with *V. dahliae* isolates WT_12253 or Δ*frq_12253* at 0, 7, 14, 21, and 28 days post-inoculation under 12:12 LD cycle. The area under the disease progression curve (AUDPC) was calculated. Different letters indicate statistical differences (*p*-value < 0.05), Tukey’s HSD test. Disease symptoms 28 days after inoculation of *A. thaliana* seedlings with *V. dahliae* isolates and a mock (control) inoculation are shown on the right. **(D)** Disease score of *A. thaliana in vitro* seedlings inoculated with *V. dahliae* isolates WT_12008 or Δ*frq_12008.* **(E)** Disease score of the susceptible strawberry cultivar Hapil inoculated with WT_12253 and Δ*frq_12253*, 6 weeks after inoculation. A one-way ANOVA was performed. Data are presented as mean (±SEM) with different letters indicating statistical differences (*p*-value < 0.05). Disease symptoms 6 weeks after inoculation of strawberry plantlets with *V. dahliae* isolates and a mock (control) inoculation are shown on the right. **(F)** Disease score of the susceptible strawberry cultivar Hapil inoculated with WT 12008 and Δ*frq_12008.* The same mock picture is shown in **(D)** and **(F)** as these plants served as controls in an experiment in which plants were inoculated with either sterile water (mock), 12253 or 12008 isolates.

Having found no strong evidence of circadian rhythmicity we wondered what function *V. dahliae* FRQ might have. To assess whether *Vdfrq* plays a role in development, we assessed whether the banding pattern of conidia and microsclerotia is generated by the *Vdfrq* knockout mutants Δ*frq_12253* and Δ*frq_12008*. The Δ*frq* mutants grown in either 12:12 h LD cycles or temperature cycles (20–28°C) present the same characteristic banding pattern of microsclerotia and conidia as the wild type (WT) strains (Figures 5A,B, LH panels). Despite the variability among replicates we observed a reduction in total colony growth for both Δ*frq* mutants (Δ*frq_12253* and Δ*frq_12008*) with respect to the WT (12253 and 12008) strains (*p*-value < 0.01, *p*-value < 0.01, respectively). The reduction in daily growth was independent of lighting conditions (Figures 5A,B) but dependent on the nutritional composition of the culture medium (Supplementary Figure S8). Δ*frq_12008* showed reduced growth compared to WT_12008 on PLYA media, but not on a minimal medium (MM) (*p-*value = 0.99), Czapek Dox agar (DOX) (*p*-value = 1) or basal modified medium (BMM) (*p*-value = 1). Colonies of Δ*frq_12253* were significantly larger than WT_12253 when incubated on BMM and MM (*p*-value < 0.01, *p*-value < 0.01, respectively). Bands of microsclerotia were observed on all media types, but conidial rings were masked by masses of mycelium when grown on DOX and BMM media (Supplementary Figure S8). Although a large heterogeneity exists between *Vdfrq* mutants of different *V. dahliae* isolates, the results suggest that *Vdfrq* plays a role in normal fungal growth.

### *V. dahliae*Δ*frq* Leads to Widespread Transcriptional Differences

Because LREs are present in the *Vdfrq* promoter and to uncover the possible function of *Vdfrq* we compared gene expression in dark-grown WT and Δ*frq* cultures after exposure to 6 h of white light. Of 11388 genes with non-zero total counts, 235 genes are up-regulated and 162 genes are down-regulated in the WT strain (L vs. D in V.d 12253). In the Δ*frq* mutant, 65 genes are up-regulated and 30 genes are down-regulated (L vs. D in Δ*frq_12253*). It is interesting that in the Δ*frq* strain, light has a reduced effect on induction/repression of gene expression suggesting that VdFRQ may indirectly affect light signaling.

The deletion of *Vdfrq* has a large effect on gene expression. Interestingly, the difference in gene expression between the Δ*frq* and the WT is greater in the light than in the dark: 278 genes are up-regulated and 195 genes are down-regulated in the Δ*frq_12253* mutant grown under dark conditions (Δ*frq* vs. WT in D), whereas 435 genes are up-regulated and 233 genes are down-regulated in the Δ*frq_12253* mutants harvested after 6 h in the light (Δ*frq* vs. WT in L).

Functional enrichment analysis was carried out to investigate the function of genes differentially expressed between the WT and the Δ*frq_12253* mutant. Regardless of light conditions Δ*frq_12253* up-regulated genes are involved in metabolic processes, translation, protein secretion and nucleotide metabolic processes. The down-regulated genes in Δ*frq*_12253 grown in either light or dark were involved in redox processes, heme oxidation, circadian rhythms, and glutamate biosynthetic processes. Additionally, genes that are down-regulated only in the light are functionally enriched for phosphate ion transport, nitrate assimilation transport, superoxide anion generation, one-carbon metabolic processes as well as pathogenesis processes (Supplementary Table S3).

### *V. dahliae frq* Regulates the Expression of Photoreceptor, TF- and SM- Encoding Genes

In *Neurospora*, the transcription factor complex of the clock oscillator machinery activates the transcription of other TFs which results in a gene regulatory cascade (Chen et al., 2009; Smith et al., 2010). As a result, the circadian clock modulates the expression of genes involved in many processes, especially those involved in physiology and metabolism (Hurley et al., 2014). Therefore, we hypothesized that disruption of *Vdfrq* would affect the expression of photoreceptors, transcription factor-encoding genes and genes involved in secondary metabolism.

We looked at how the absence of VdFRQ affects expression of clock, photoreceptor, transcription factor and secondary metabolism-encoding genes in *V. dahliae* under different light conditions (6 h dark and 6 h light). The expression of *Vdfwd-1*, Vd*wc-1*, and Vd*wc-2* are significantly down-regulated in Δ*frq_12253* in both light and dark conditions, although the LFC in expression does not reach the threshold of 1 (Supplementary Table S4). Interestingly, when exposed to light *vvd* is significantly up-regulated in the Δ*frq_12253* mutant, indicating that VdFRQ has a negative effect on *vvd* transcription. In the case of *cry-dash* gene, its expression is light-regulated in both the WT and Δ*frq_12253*, although the absolute expression level in the *Vdfrq* mutant is significantly lower than in the WT. The expression profile of the *rgs-lov*, *cry-1*, *phy*, *phr*, and *nop-1* genes does not change in the absence of *Vdfrq*.

We found that the absence of *Vdfrq* also affects the expression of several TF-encoding genes. A total of 50 genes containing functional annotations associated with TFs exhibited differences in expression due to the lighting conditions or strain background. Most of the differences in expression were due to the *Vdfrq* mutation. 26 TF-encoding genes were up-regulated, and 8 TF-encoding genes were down-regulated in Δ*frq_12253* regardless of the light conditions. However, there were 6 TF-encoding genes that were no longer light-induced or light-repressed in the Δ*frq_12253* mutant strain (Supplementary Table S4).

Deletion of *Vdfrq* also affected core secondary metabolite-biosynthetic gene expression. The lovastatin non-aketide synthase encoding gene (VDAG_JR2_Chr1g23880) (Yun et al., 2015) alongside 7 other members of the PKS gene cluster (cluster 14) are strongly affected in the absence of *Vdfrq*. Interestingly, the light-induced expression of these genes was compromised in Δ*frq_12253* (Supplementary Table S5). Additionally, 6 genes of cluster 14 are the top most down-regulated genes in Δ*frq_12253* (LFC between −1.5 and −3.4), including the lovastatin non-aketide synthase encoding gene, TOXD protein-encoding gene and a hydrolase encoding gene. An additional cluster of PKS encoding genes, the putative aflatoxin biosynthetic cluster (cluster 17), exhibit 10 up-regulated genes in the absence of *Vdfrq*. Several members of cluster 24 (PKS), where the core biosynthetic gene encodes a fatty acid synthase, also exhibit overexpression in Δ*frq_12253*. Furthermore, 4 genes of the 5 non-ribosomal peptide synthases (NRPS, cluster 78) are highly up-regulated (LFC > 2) in the absence of *Vdfrq* (Supplementary Table S5). Therefore, *Vdfrq* is crucial for the regulation of expression of secondary-metabolism-encoding genes in *V. dahliae*.

### *V. dahliae* Δ*frq* Mutants Display Reduced Pathogenicity in a Strain-Dependent Manner

In *Botrytis cinerea* the circadian clock regulates virulence (Hevia et al., 2015, 2016) and consistent with this finding, in a small number of fungi, circadian clock mutants show altered pathogenicity. To assess whether the loss of *Vdfrq* would influence the process of infection we evaluated pathogenicity of two wild-type isolates (*V. dahliae* 12253 and 12008) as well as Δ*frq_12253* and Δ*frq_12008* on *A. thaliana* and *Fragaria*× *ananassa in vitro*-grown plants. Both wild type isolates were isolated from United Kingdom strawberries and fall within the VC group subclade II-2, 12008 being a highly virulent isolate and 12253 being a moderately virulent isolate. The infected seedlings were incubated in a 12:12 LD cycle for 28 days. Symptoms were visually rated at 0, 7, 14, 21, and 28 dpi on a scale of 1–9, in which 1 was equal to no symptoms and 9 equaled a dead plant. In *A. thaliana* seedlings infected with the wild-type strain WT_12253 present symptoms in up to 75% of the leaves after 21 days of inoculation (Figure 5C). At the same time post inoculation plants infected with Δ*frq_12253* show symptoms of wilt on 20% of leaves. The difference is more obvious at 28 dpi, when most plants infected with WT_12253 are dead whilst Δ*frq_12253* infected plants display slight chlorotic symptoms in several outer leaves. The AUDPC confirmed a significant difference in pathogenicity between Δ*frq_12253* and WT_12253 strains (*p*-value < 0.01). Contrary to this observation, WT_12008 and Δ*frq_12008* do not present differences in virulence (Figure 5D). Similar results are obtained from pathogenicity tests on a susceptible strawberry cultivar (Hapil). After 6 weeks of inoculation, plantlets infected with the Δ*frq_12253* strain show fewer disease symptoms than the WT_12253 strain (Figure 5E), whereas the WT_12008 and Δ*frq_12008* strains do not show differences in the ability to cause disease (Figure 5F). These results indicate that pathogenicity of the *Vdfrq* mutant is impaired in an isolate-dependent manner, with isolates that are more pathogenic showing no significant reduction in pathogenicity when *frq* is deleted.

## Discussion

Evidence that the outcome of a plant-pathogen interaction can depend on the time of day at which the interaction occurs (Bhardwaj et al., 2011; Wang et al., 2011; Zhang et al., 2013), has recently put the spotlight on the study of the circadian clock in plant pathogenic fungi. There are multiple examples of important fungal species harboring the genetic components of the circadian clock, but the role of the clock on pathogenicity is not well understood. Optimizing the processes of infection to be in synchrony with a plant’s most susceptible time could be advantageous to some fungal species for more efficient infection. Similarly, understanding the daily changes in the developmental stages of pathogenic organisms could help design more precise disease control strategies in agriculture.

In agreement with the findings of Salichos and Rokas (2010), core clock orthologs were found in most of the tested Sordariomycetes species, including important plant-pathogenic fungi. Homologs of clock genes were identified in all species of the *Verticillium* genus: *V. albo-atrum*, *V. alfalfae*, *V. nonalfalfae*, *V. dahliae*, *V. longisporum* subgenome D, *V. nubilum*, *V. tricorpus*, *V. isaacii*, *V. klebahnii*, and *V. zaregamsianum.* This result contrasts with previous analysis in which loss of the *wc-2* homolog in *V. albo-atrum* was reported (Salichos and Rokas, 2010), probably due to the poor quality at this time of the publicly available *V. albo-atrum* genome. Several species of the Dothideomycetes and Leotiomycetes, such as *Blumeria graminis*, *Cercospora zeae-maydis*, *Alternaria alternata*, and *Venturia inaequalis* lack a homolog of the blue-light receptor *vvd*, but do contain homologs of the other clock components.

The *V. dahliae* core clock homologs display strong conservation at the domain level. Remarkably, *Vd*FRQ contains all the domains identified in *N. crassa* FRQ and shares similar NLS sequences required for import of the protein to the nucleus. In addition, *Vd*FRQ exhibits conservation of phosphorylation sites, crucial for regulated activity and degradation and that determine periodicity in *N. crassa* (Liu et al., 2000). However, small changes in domains may be crucial for function. For instance, although a coil–coil domain is present in *Vd*FRQ the probability that it can form dimers is much lower than that of the *N. crassa* FRQ coil–coil domain. Without dimerization FRQ does not interact with the WHITE-COLLAR proteins and overt rhythmicity is lost (Cheng et al., 2001). Proteins forming the WCC are also conserved in *V. dahliae*, but *Vd*WC-1 lacks the C-terminal polyQ region and has lost conservation at the N-terminal. The N-terminal of WC-1 contains important domains required for protein–protein interaction and subsequent transcriptional activation in *N. crassa* (Wang et al., 2014). Nevertheless, zinc fingers and basic regions of both WC-1 and WC-2 required for binding DNA are present and WCC phosphosites that govern circadian repression in Neurospora (Liu et al., 2000) are conserved. Thus, *V. dahliae* contains all the components required for a TTFL but changes in some important domains may compromise their ability to generate an oscillator.

In *Neurospora*, the WCCs that activate transcription in response to light and rhythmically in the dark differ in composition, in the DNA motifs they bind, and in the regions of the WCC proteins required for DNA binding. The WCC that responds to light is composed of two WC-1 proteins and one WC-2 protein. The complex binds close to the transcriptional start site of *frq* and binding requires only the zinc finger and proximal basic region of WC-2. In contrast the heterodimer of WC-1 and WC-2, responsible for transcriptional activation of *frq* in the dark, binds to the clock-box over 1 kb upstream of the transcriptional start site. Binding to the DNA requires zinc fingers and basic regions of both proteins and recruitment of chromatin modifiers SWI/SNF to initiate transcription (Wang et al., 2016). Although several putative LREs were identified in the *Vdfrq* promoter, core sequences of the clock box that have previously been found within 1–3 bp of each other, in *V. dahliae* are separated by 80 bp. The lack of a classic clock box may underlie the lack of circadian rhythmicity of *Vdfrq* expression. However, ChIP-Seq studies have revealed diverse motifs bound by light-activated WCC (Smith et al., 2010) and Chen et al. (2012) report that the human GATA3 protein can bind palindromic GATA sites and GATA sites located on different molecules of DNA, indicating that perhaps proximity of binding motifs is not necessarily limiting.

In order to determine whether the *V. dahliae* morphological rhythm was under the control of a circadian clock, a variety of tests were performed. Although rhythms of conidiation and microsclerotia development are observed under light-dark and temperature cycles, they do not persist in the absence of external stimuli and thus lack a key characteristic of circadian rhythms (Pittendrigh, 1960). This result, repeated on different media, supports the tentative conclusion that the lack of sustainable developmental rhythms in constant conditions is unlikely to be due to media composition. Possible explanations for the absence of observable clock-controlled free-running developmental rhythms in *V. dahliae* include dampening of an existing rhythm in the absence of external signals or the lack of a functional clock.

If the former is true we theorized that the existence of a circadian clock could be revealed through analysis of development under different entrainment regimens. Frequency demultiplication effects whereby clock-controlled outputs occur once every 24 h when external periods are close to half of the endogenous period (*T* = 12) are observed in circadian rhythms (Pittendrigh, 1960; Greene et al., 2003). Exposure to long periods (*T* = 48) have the opposite effect, resulting in a reduction in the frequency of the output to once every 24 h (Pittendrigh, 1960; Yoshida et al., 2008). *V. dahliae* grown under 6:6 LD or 28:28 LD cycles did not exhibit frequency demultiplication and produced rings of development every 12 or 56 h, respectively.

A defining characteristic of circadian clocks is their ability to entrain to external stimuli such as light and temperature (Pittendrigh, 1960). In *N. crassa*, short pulses of light trigger a rapid induction of *frq* transcription that result in the resetting of the clock (Crosthwaite et al., 1995). WC-1 is required for photoinduction of *frq* in response to light not only in *N. crassa* (Froehlich et al., 2002) but in other fungal species (Hevia et al., 2015; Traeger and Nowrousian, 2015; Franco et al., 2017). However, our results show that whereas *V. dahliae* photoreceptor-encoding genes *Vdvvd* and *Vdcry-dash* rapidly respond to light, *Vdfrq* expression is not light-induced. Furthermore, *Vdfrq*, *Vdwc-1*, *Vdwc-2*, *Vdvvd* and *Vdccg-16* transcript levels do not show robust anticipatory behavior nor the significant rhythmicity in light or temperature cycles seen in other fungi with circadian clocks (Merrow et al., 1999; Hevia et al., 2015; Traeger and Nowrousian, 2015). Constitutive expression of *Vdfrq* under cyclic environmental conditions could be a symptom of a dysfunctional FRQ-WC clock. Alternatively, if a post-transcriptional FRQ-WC clock runs in *V. dahliae* it may represent an ancestral oscillator that in some fungi has subsequently been reinforced through additional feedback regulation acting on transcription and mRNA abundance.

RT-PCR analysis of *V. dahliae* gene expression in constant darkness after light entrainment revealed no circadian oscillation of *Vdfrq* mRNA in the isolates tested. In addition, RNA-seq gene expression studies over a 24-h period revealed no indication of strong rhythmic expression of transcripts that in other fungi are clock-associated.

With the exception of *ccg-16* mRNA, the difference between the highest and lowest levels of these clock-associated and photoreceptor transcripts is never more than 2 log_2_-fold change and in the majority of transcripts considerably less than 1 log_2_-fold change. If circadian rhythms in mRNA abundance are indeed present in *V. dahliae*, high resolution sampling over a 48-h time-course and increased replication of experiments will be needed to reveal the very low amplitude changes that occur (Li et al., 2015). In conclusion, we observed no strong signature of rhythmic gene expression that would indicate possible regulation of mRNA levels by a circadian clock in *V. dahliae*.

In order to determine the impact of *Vdfrq* on the morphology of *V. dahliae* it was deleted in two different isolates. The absence of *Vdfrq* does not lead to the abolishment of developmental rhythms but results in reduced colony growth on most media. Moreover, pathogenicity tests reveal reduced infectivity of *Vdfrq* mutants of a weakly pathogenic isolate but normal disease progression of a highly virulent isolate. Interestingly, this observation is repeatable across plant species (*A. thaliana* and *Fragaria*× *ananassa*). These data suggest that the growth penalty and/or specific changes in the expression of genes unrelated to growth in the *Vdfrq* deletion mutants influence infection and disease symptoms in a strain-dependent manner. Further study of a wide range of isolates will be needed to determine if the influence *Vdfrq* has on pathogenicity is correlated with virulence of the wildtype parent. Transcriptional profiling of a *Vdfrq* knockout mutant revealed possible roles for VdFRQ in metabolic and signaling processes and in pathogenicity. This result is in agreement with the observation that circadian control has a major impact on metabolism in *N. crassa* (Hurley et al., 2014). Interestingly, the absence of *Vdfrq* has an effect on the light response. One reason for this could be that in Δ*Vdfrq* expression of *Vdwc-1* is down-regulated. This prompts speculation that a *Vdwc-1* deletion would also affect pathogenicity. To summarize, our data reveal large changes in gene expression, altered growth and pathogenicity in the *Vdfrq* deletion mutant. Whether or not these phenotypes result from *Vd*FRQ functioning outside of a circadian clock cannot at present be ascertained.

With regard to the existence of a circadian clock in *V. dahliae* our results suggest three possibilities; (i) the clock is absent, (ii) the clock is post-transcriptional and constitutive gene expression leads to oscillation at the protein level, (iii) the clock is only active during specific developmental stages and/or specific conditions, e.g., the clock is activated when a host is detected and is only functional *in planta*. As *V. dahliae* infects and moves through host tissue it is likely that an ability to anticipate time-of-day changes in host immunity would be beneficial.

At least three circumstances can be envisaged where circadian rhythmicity might be absent. The first is when an organism is always ready to respond to the rhythmic environment. It has been reported that despite the presence of homologs of most clock genes in *Picea abies* (Norway Spruce) Gyllenstrand et al. found no evidence of circadian gene expression in constant conditions (Gyllenstrand et al., 2014). The authors note that because gymnosperms make chlorophyll in the dark the strong adaptive pressure to anticipate dawn is lacking. Indeed, there is little evidence to support circadian gene transcription/expression in gymnosperms (Oberschmidt et al., 1995; Piechulla et al., 2001). Nevertheless, night break experiments indicate that a circadian rather than an hour-glass clock is used in photoperiodism (Clapham et al., 2001; Ekberg et al., 2010; Pokhilko et al., 2012). The second circumstance is in an environment where the absence of a circadian clock reduces the organism’s ability to anticipate and respond to a changing environment but this has no adverse effect. In a mutant form of einkorn wheat, rhythmicity of known clock and clock-regulated genes is lost. Counter-intuitively, rather than having a detrimental effect, in certain environments this mutant is more productive and less variable than the wild-type (Gawroński et al., 2014). Importantly, the presence of an alternative circadian clock running under these conditions cannot be ruled out. The third circumstance is in a predominantly arrhythmic environment, for example in underground caves and burrows where changes in temperature and humidity are minimal, or during the long winter night and perpetual daylight during mid-summer at high latitudes. Some organisms living under such conditions on initial inspection have indeed shown little or no evidence of circadian rhythmicity however, when studied in more detail these early conclusions have been overturned (Yerushalmi and Green, 2009).

While definitive proof of the absence of a circadian clock is difficult to obtain, the evidence for post-transcriptional clocks and the importance of post-transcriptional modification of clock proteins is abundant and strong. A classic demonstration that post-transcriptional processes can generate a circadian clock was provided by Nakajima et al. (2005) who showed that cyanobacterial clock proteins KaiA, KaiB, and KiaC *in vitro* in the presence of ATP exhibit cycles of phosphorylation and dephosphorylation that have a period of approximately 24 h, are self-sustainable and temperature compensated (Nakajima et al., 2005; Tomita et al., 2005). Many post-transcriptional processes act on clock gene transcripts and proteins and are key to the generation of circadian rhythmicity (Mateos et al., 2018). Indeed, it is long known that rhythms persist in enucleated *Acetabularia crenulata* (Sweeney and Haxo, 1961) but there are also numerous examples of rhythmically expressed animal and plant clock genes that when constitutively expressed do not ablate rhythmicity. Rather post-transcriptional mechanisms maintain rhythmic expression and activity of the clock proteins (Hastings et al., 2008; O’Neill et al., 2011), i.e., rhythmic transcription enhances the amplitude of rhythmic post-transcriptional processing. Indeed, even some rhythms generated post-transcriptionally are not necessarily essential parts of circadian clocks. For example, in Neurospora circadian rhythms of FRQ abundance can be decoupled from its activity (Larrondo et al., 2015).

The results in this study demonstrate conservation of the core clock proteins between *V. dahliae* and *N. crassa*. However, rhythmic gene expression in *V. dahliae* was not detected in either LD or free-running conditions. Thus, if a circadian clock is absent in *V. dahliae* then, at least in this fungus, the other function(s) of *Vd*FRQ must require a very similar domain structure. On the other hand, if constitutive levels of mRNA give rise to a solely protein-based circadian clock in *V. dahliae* our data also indicate that rhythmic outputs are not regulated at the level of mRNA abundance. An alternative possibility is that generation of circadian rhythmicity in *V. dahliae* is conditional on specific environmental conditions. The recent characterization of a *frq*-dependent circadian oscillator in the Leotiomycete *Botrytis cinerea* suggests that *frq* is a component of circadian oscillators in fungal groups that evolved concurrently with *N. crassa* (Hevia et al., 2015; Traeger and Nowrousian, 2015). By extrapolation, when *frq* and other key clock genes are represented in a genome the expectation is that a FRQ-WC clock is present. This is true even when no overt rhythms in behavior or development can be detected because clock-regulated timing of cellular biochemistry can confer a competitive advantage (Ouyang et al., 1998; Dodd et al., 2005). In the wild *V. dahliae* microsclerotia germinate in the presence of root exudates (Heale and Isaac, 1965) and it is possible that this signal initiates oscillations of a circadian clock. Future studies will determine whether or not a circadian clockwork emerges *in planta* and if so what advantages this confers on the *V. dahliae* infection cycle.

## Author Summary

Circadian clocks are used by organisms to orchestrate the activity of cellular processes such that they occur at an optimal time of day. Research carried out in the filamentous fungus *Neurospora crassa* has revealed a huge amount of information about the components of its circadian clock, its interactions with the environment and how it drives cellular biochemistry and physiology. Although homologs of the Neurospora clock genes are present in a number of fungi, functional clocks have been demonstrated in just a handful. Importantly, a link between the circadian clock of the plant pathogen *Botrytis cinerea* and virulence has recently been reported. We report that another significant plant pathogen, *Verticillium dahliae*, contains well-conserved homologs of all key clock genes. We find that diurnal development of conidia and microsclerotia is not influenced by a circadian clock. Furthermore, in a constant environment we find no evidence of rhythmic transcript accumulation. However, deletion of the *frequency* gene, that in Neurospora encodes a central clock component, results in altered growth and reduced virulence. This led us to question the role of clock genes in Verticillium. We are forced to consider that in this species the interactions that generate rhythmicity have been lost, are generated purely via post-transcriptional modification of clock proteins, are only triggered when specific environmental conditions are met or never evolved.

## Data Availability Statement

The original contributions presented in the study are publicly available. This data can be found here: https://www.ncbi.nlm.nih.gov/PRJEB39510.

## Author Contributions

EC-L and RH designed the experiments. EC-L performed the experiments. EC-L, SC, LJ, and RH analyzed the data and wrote and edited the manuscript. All authors contributed to the article and approved the submitted version.

## Conflict of Interest

The authors declare that the research was conducted in the absence of any commercial or financial relationships that could be construed as a potential conflict of interest.

## Footnotes

1 https://expressionanalysis.github.io/ea-utils/

## Supplementary Material

FIGURE S1 Generation of *V. dahliae*Δ*frq* mutants. **(A)** Strategy followed to replace *V. dahliae frq* with *hygromycin phosphotransferase*. Genomic regions utilized for homologous recombination (black fragments) are shown. Black arrows symbolize primer pairs used for the validation PCR. **(B)** Representative gel of the validation PCRs for the correct *V. dahliae* knockout transformants. **(C)** Primer pairs utilized in PCR validation.

FIGURE S2 **(A)** Alignment of FRQ sequences from *N. crassa* and *V. dahliae* JR2. Protein domains characterized in *N. crassa* FRQ are annotated in red: Coiled-coil domain, FCD, FFD and PEST domains. Green bar, nuclear localization signal (NLS). Blue circle, conserved serine residue at position 513 (Ser-513). Green circles, additional phosphorylation sites previously identified in *N. crassa*. **(B)** Multiple *frq* alignment of several *V. dahliae* strains. The alignment includes *V. dahliae* of vegetative compatibility (VC) subclade II-1 (JR2, 12008, 12253, and 12251) and VC subclade II-2 (12161 and 12158). Several single nucleotide polymorphisms (SNPs) are observed between species from VC subclade II-1 and VC subclade II-2. The SNPs are marked in colors where purple is the nucleotide C, yellow is G, red is A, green is T. The SNPs occur in positions 54, 105, 236, 303, 477, 1241, 1266, 1291, 1518, 1938, 2198, 2388, 2434, 2745, and 2745 of the *Vdfrq* CDS.

FIGURE S3 Alignment of *N. crassa* and *V. dahliae* WC-1. Protein domains: Dark blue poly-glutamine stretch sequences. Blue, LOV domain with black box indicating the GXNCRFLQ motif. Red, PAS domains. Yellow, predicted nuclear localization signal (NLS). Green, GATA-type zinc-finger.

FIGURE S4 Alignment of *N. crassa* and *V. dahliae* WC-2. Protein domains: Red, PAS domain. Yellow, NLS (*V. dahliae* only). Green GATA-type zinc -finger.

FIGURE S5 The morphological rhythms of 12 *V. dahliae* isolates do not free- run. *V. dahliae* strains isolated from multiple hosts were point- inoculated on PLYA plates and incubated for 14 days in an alternating 12 h dark/12 h white-light cycle (12:12 LD) (row 1) or under 12 h at 20°C/12 h at 28°C (12:12 20/28) (row 3). Plates were transferred to constant darkness (12:12 LD – DD) (row 2) or constant temperature (12:12 20/28 −Ct 24) (row 4) for 7 days following the initial 14-days incubation in cyclic environments.

FIGURE S6 The morphological rhythm of *V. albo-atrum* isolate 11001 and 11006, *V. nubilum* isolate 15001 and *V. tricorpus* isolate 20001 do not free run. The isolates were incubated on PYLA plates for 14 days under alternating 12 h white-light/12 h dark cycles (12:12 LD) (row 1) or under 12 h at 20°C/12 h at 28°C (12:12 20/28) (row 3). Plates were transferred to constant darkness (12:12 LD – DD) (row 2) or constant temperature (12:12 20/28 −*C*t 24) (row 4) for 7 days following the initial 14-days incubation in cyclic environments. Red lines indicate the period of growth under constant conditions.

FIGURE S7 Schematic representation of *frq* promoter and LRE motifs in *N. crassa*, *M. poae*, *Verticillium* spp. and *B. cinerea*. The confirmed distal LRE (dLRE) and proximal LRE (pLRE) motifs in the promoter and the qrf LRE (qLRE) motif in the terminator of *N. crassa frq* gene are marked with arrows. The putative promoter motifs containing the sequence 5′GATNC–CGATN3′He and Liu (2005) in the promoters (2,000 bp upstream the 5′ UTR) and terminator (2,000 bp downstream the 3′ UTR) are shown in blue. The 5′GATCGA3′ (Smith et al., 2010) sequences are displayed in purple.

FIGURE S8 *Verticillium dahliae* growth and developmental phenotypes. **(A)** Morphological phenotype of WT_12253, WT_12008, Δ*frq_12008*, Δ*frq_12253* strains were incubated on PLYA, Czapek DOX, MM, and BMM plates under 12:12 LD conditions for 14 days. Growth media composition affects growth rates of WT and clock mutant strains. Quantification of colony size of the strains presented in plate pictures. **(B)** Boxplots represent distribution of colony diameters after 14 days of incubation in 12:12 LD cycles. Two independent experiments, each containing three replicates. Analysis of variance (ANOVA) was performed. Letters indicate significant differences (*p*-value < 0.05) for each media type, Tukey’s HSD test.

TABLE S1 List of fungal strains. ^∗^Unknown data is labeled as Not Available (NA).

TABLE S2 List of primers.

TABLE S3 List of significantly enriched GO terms related to biological processes in Δ*frq_12253* versus the WT_12253 strain in light and dark.

TABLE S4 Expression of putative core clock genes, photoreceptor- and TF-encoding genes in WT 12253 and Δ*frq_12253* in both light and dark conditions. Transcripts displaying a Log fold change (LFC) > 1 were classified as pink, dark red if the LFC > 2, light green if the LFC < −1 and dark green if the LFC < −2. Yellow boxes indicate *p*-values < 0.05.

TABLE S5 Most differentially expressed genes in Δ*frq_12253.* Transcripts displaying a Log fold change (LFC) > 1 were classified as pink, dark red if the LFC > 2, light green if the LFC < −1 and dark green if the LFC < −2. Yellow boxes indicate *p*-values < 0.05.
