# Supplementary material for: No Evidence That Homologs of Key Circadian Clock Genes Direct Circadian Programs of Development or mRNA Abundance in Verticillium dahliae
Source: Front Microbiol. 2020 Aug 28;11:1977. doi: 10.3389/fmicb.2020.01977 (PMC7493669; doi:10.3389/fmicb.2020.01977)

Suppl. Figure 1

A

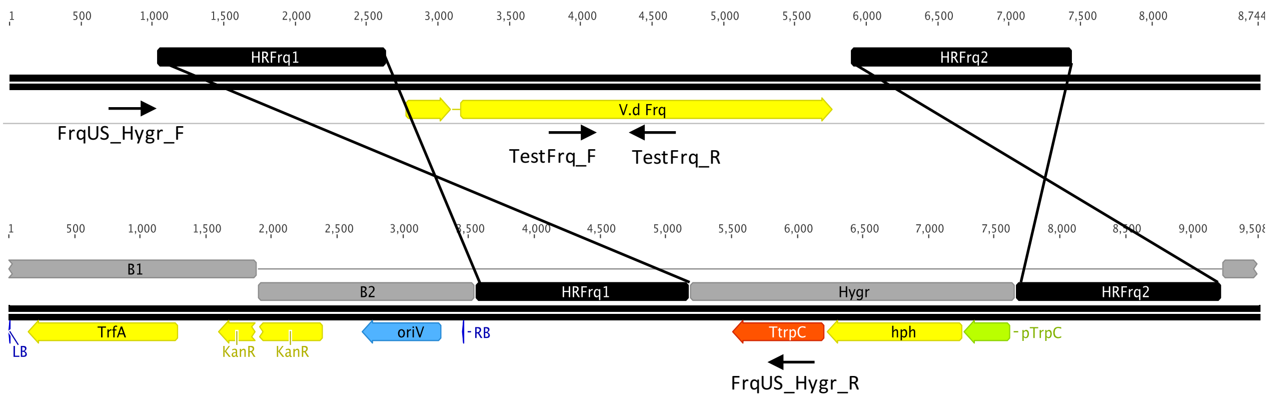

B

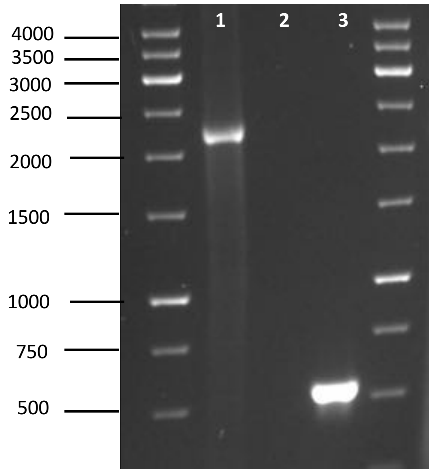

C

Primer pair	Sequence 5' - 3'	Gel lane	Size (bp)
FrqUS_Hygr	AGTTCCACTCGTTCGCTCTG	1	2182
	CGCCTATATCGCCGACATCA		
TestFrq	CCATCTTCGGCGCATTTGAG	2, 3	563
	ACTGTGAGGAATTGCTGCGA		

Suppl. Figure 2

A

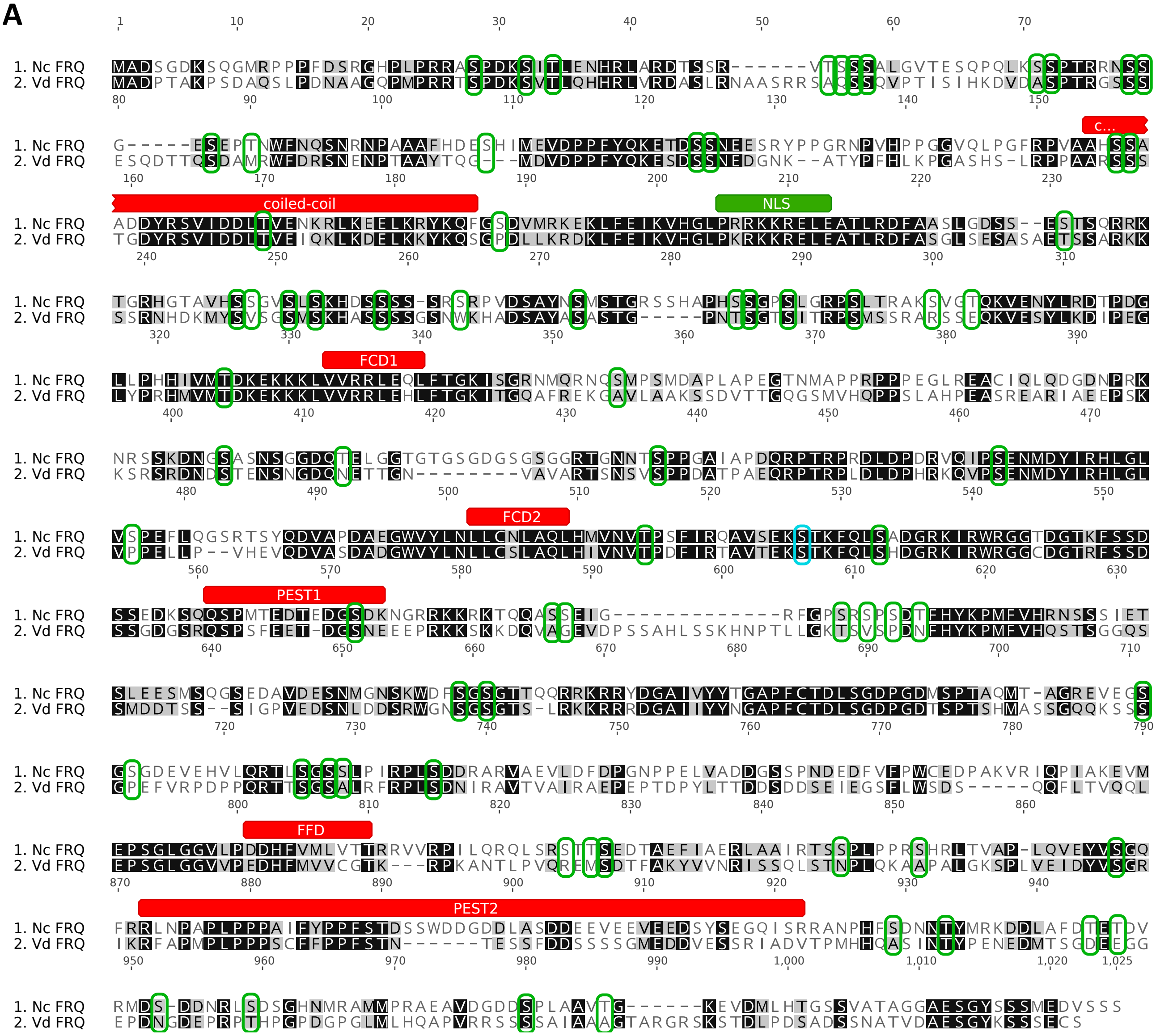

B

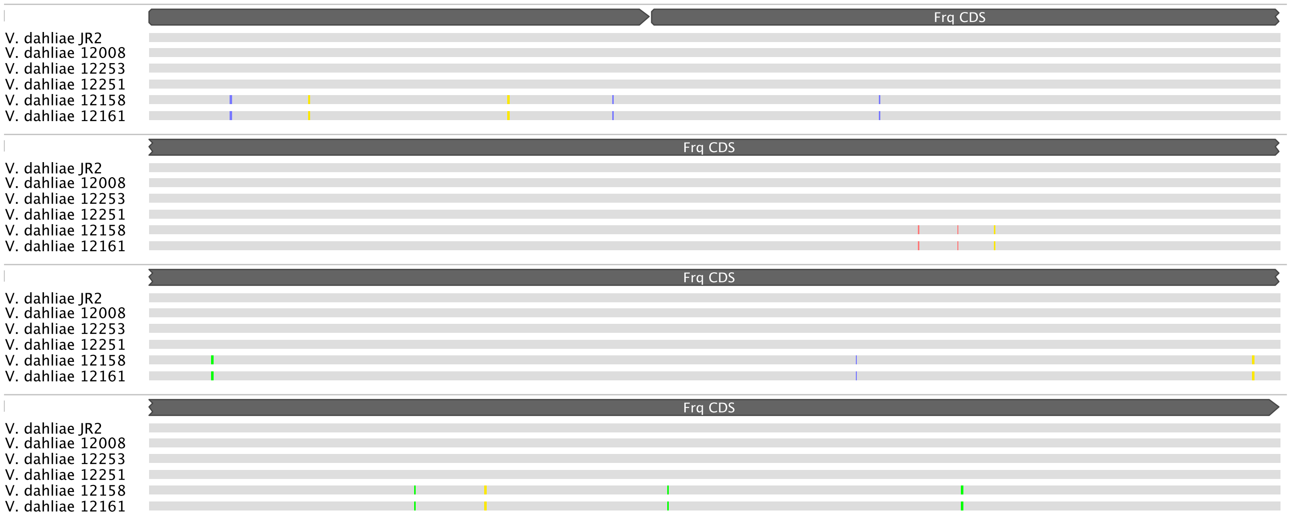

Suppl. Figure 3

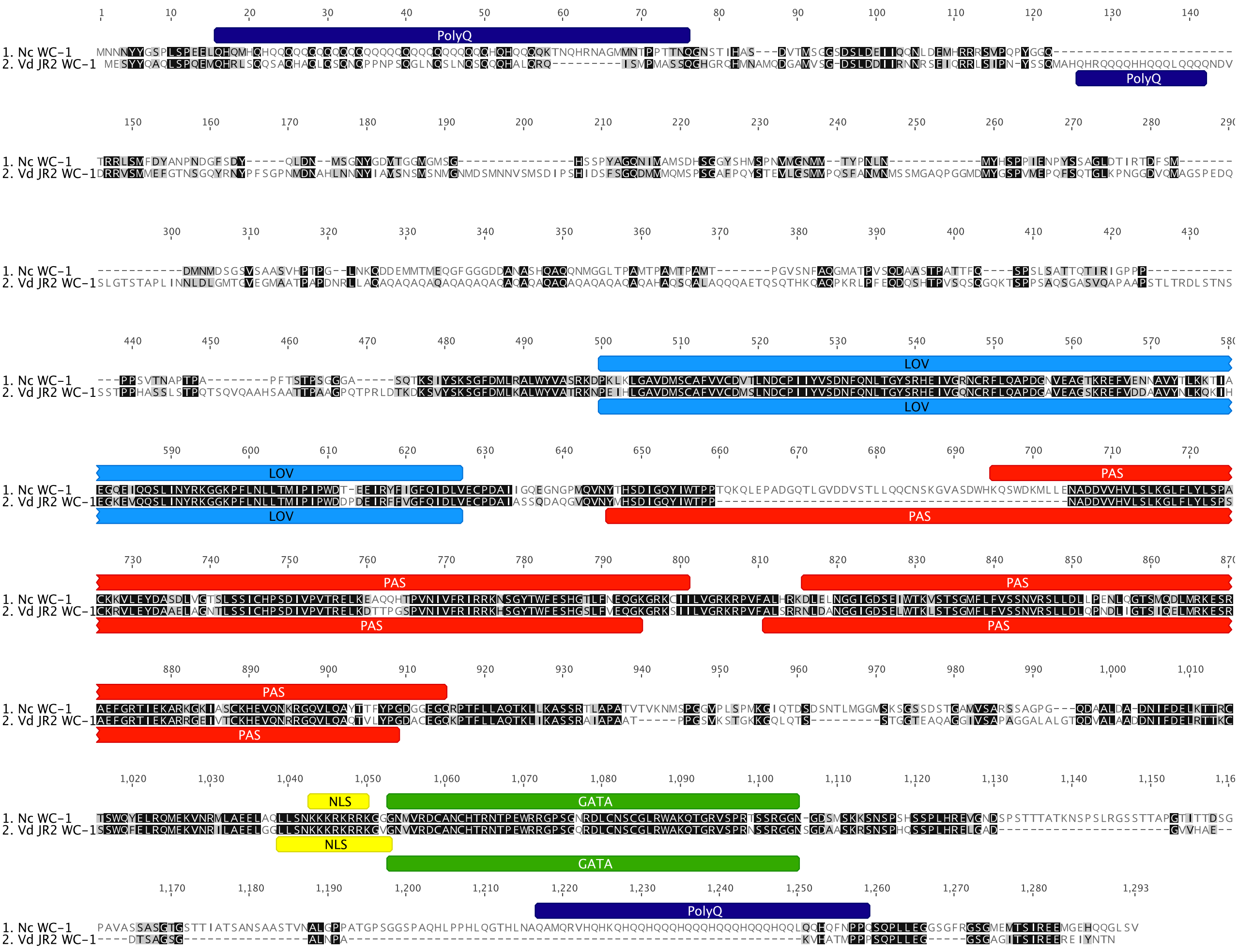

Suppl. Figure 4

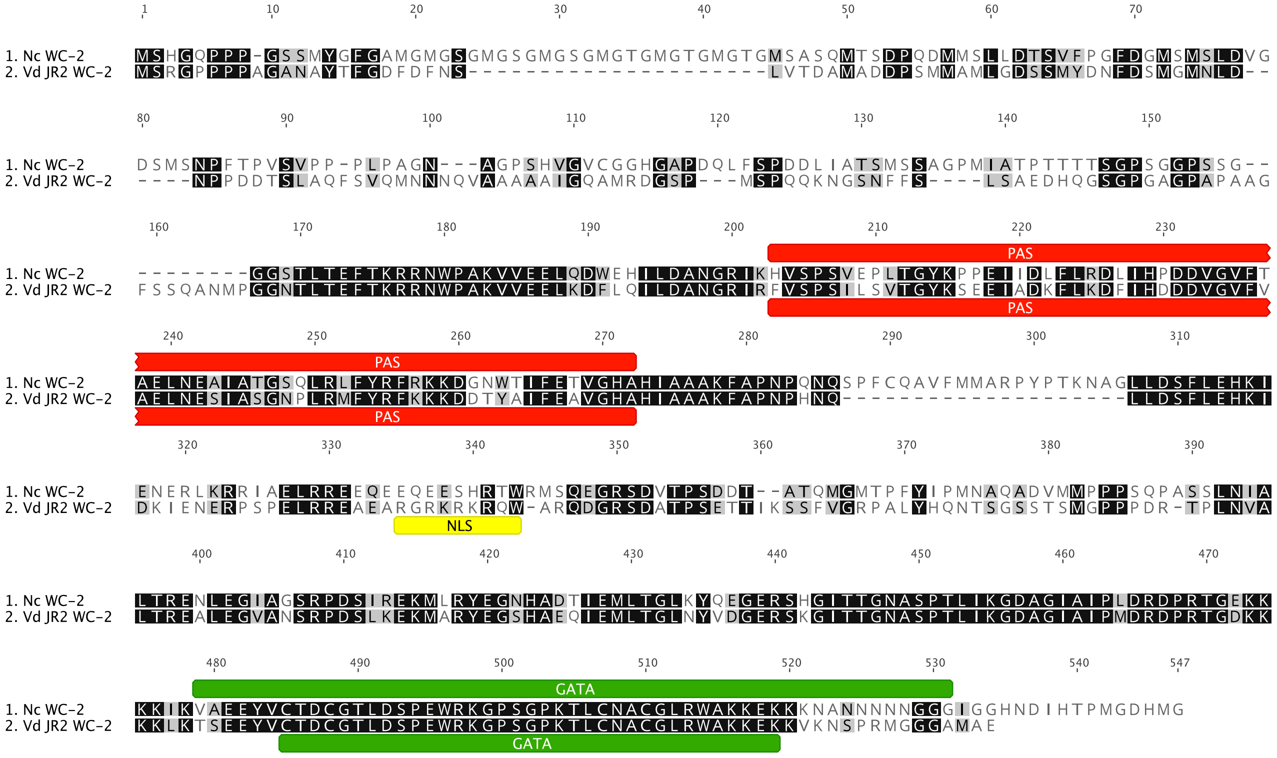

Suppl. Figure 5

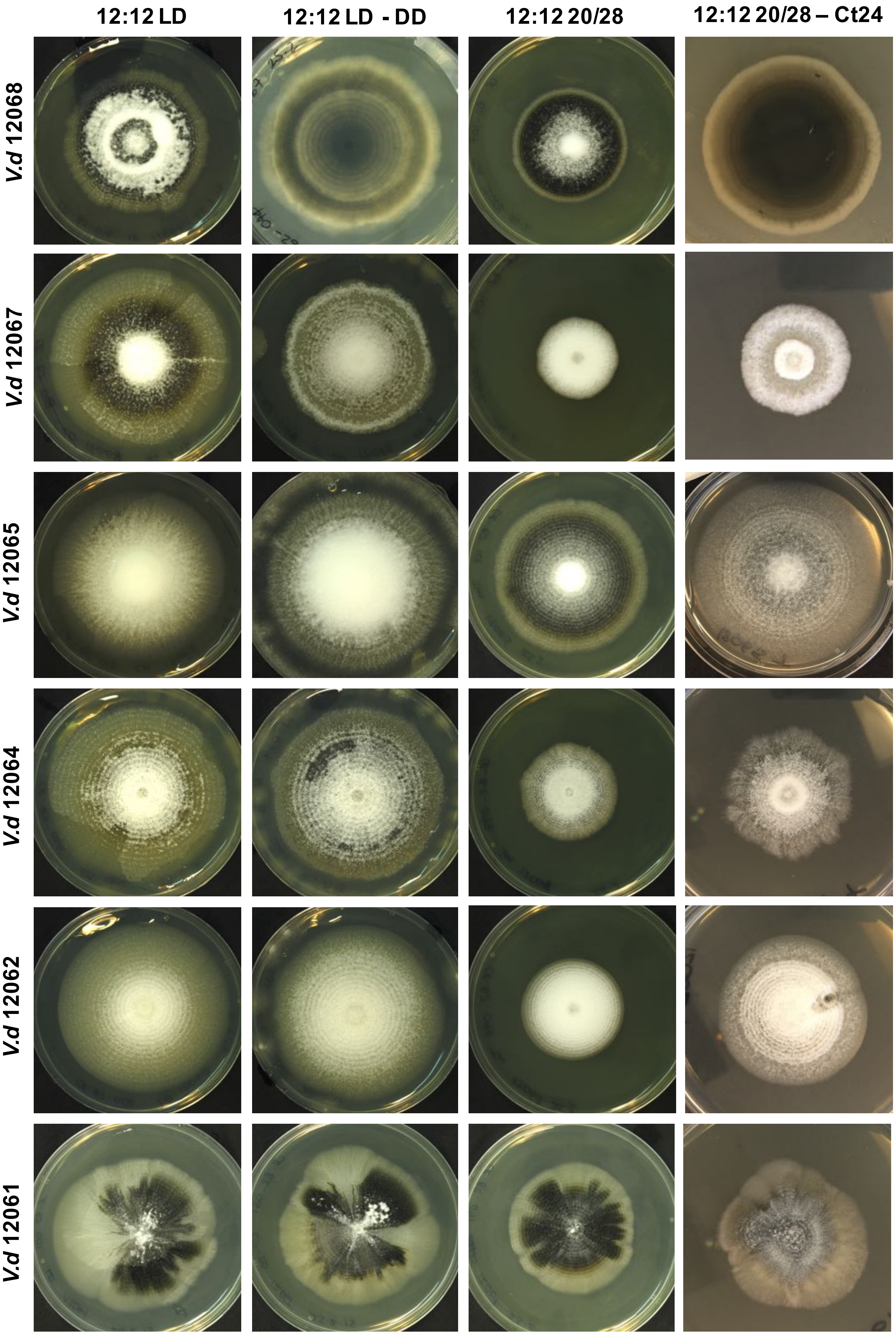

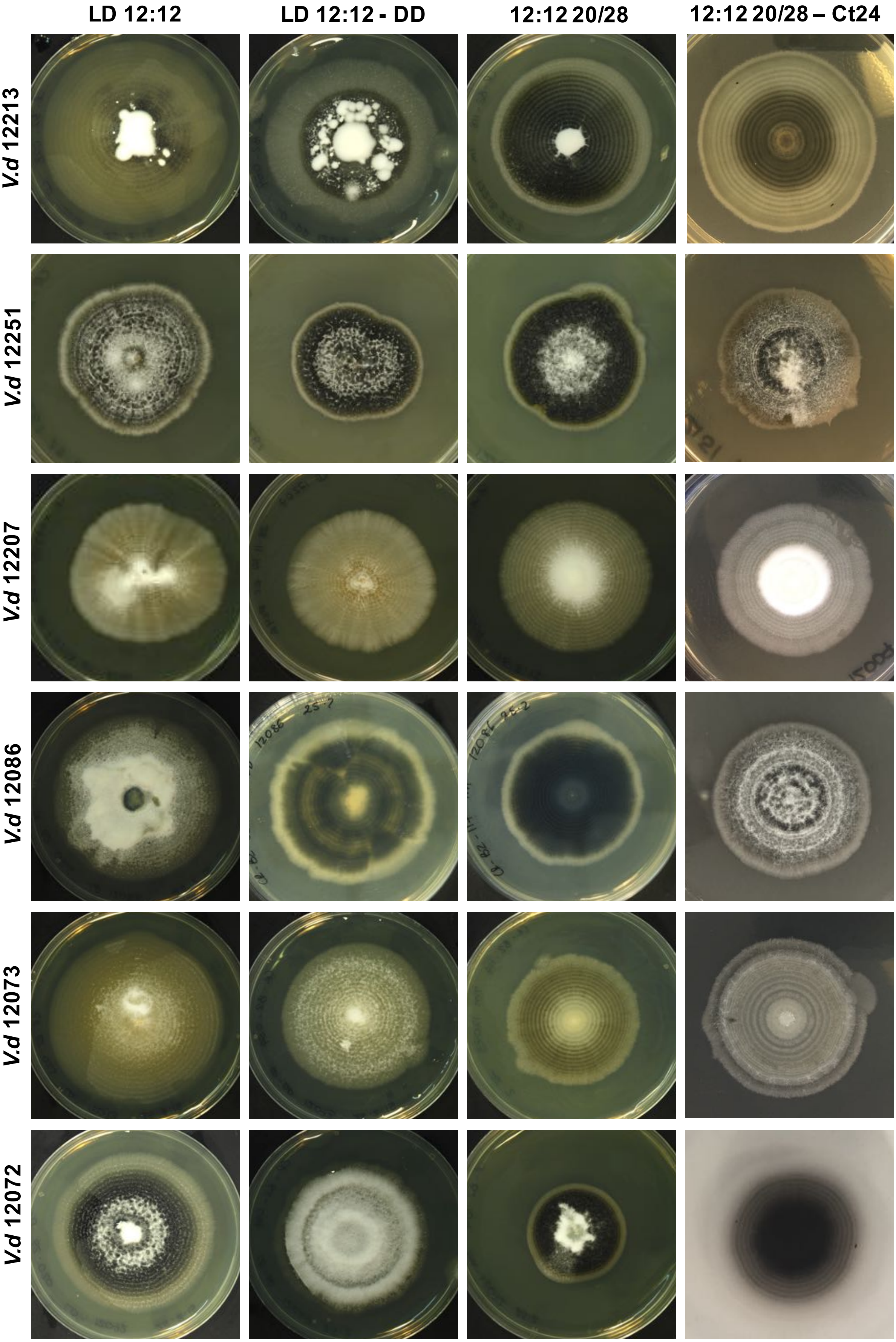

LD 12:12
LD 12:12 - DD
12:12 20/28
12:12 20/28 – Ct24
V.d 12213
V.d 12251
V.d 12207
V.d 12086
V.d 12073
V.d 12072

Suppl. Figure 6

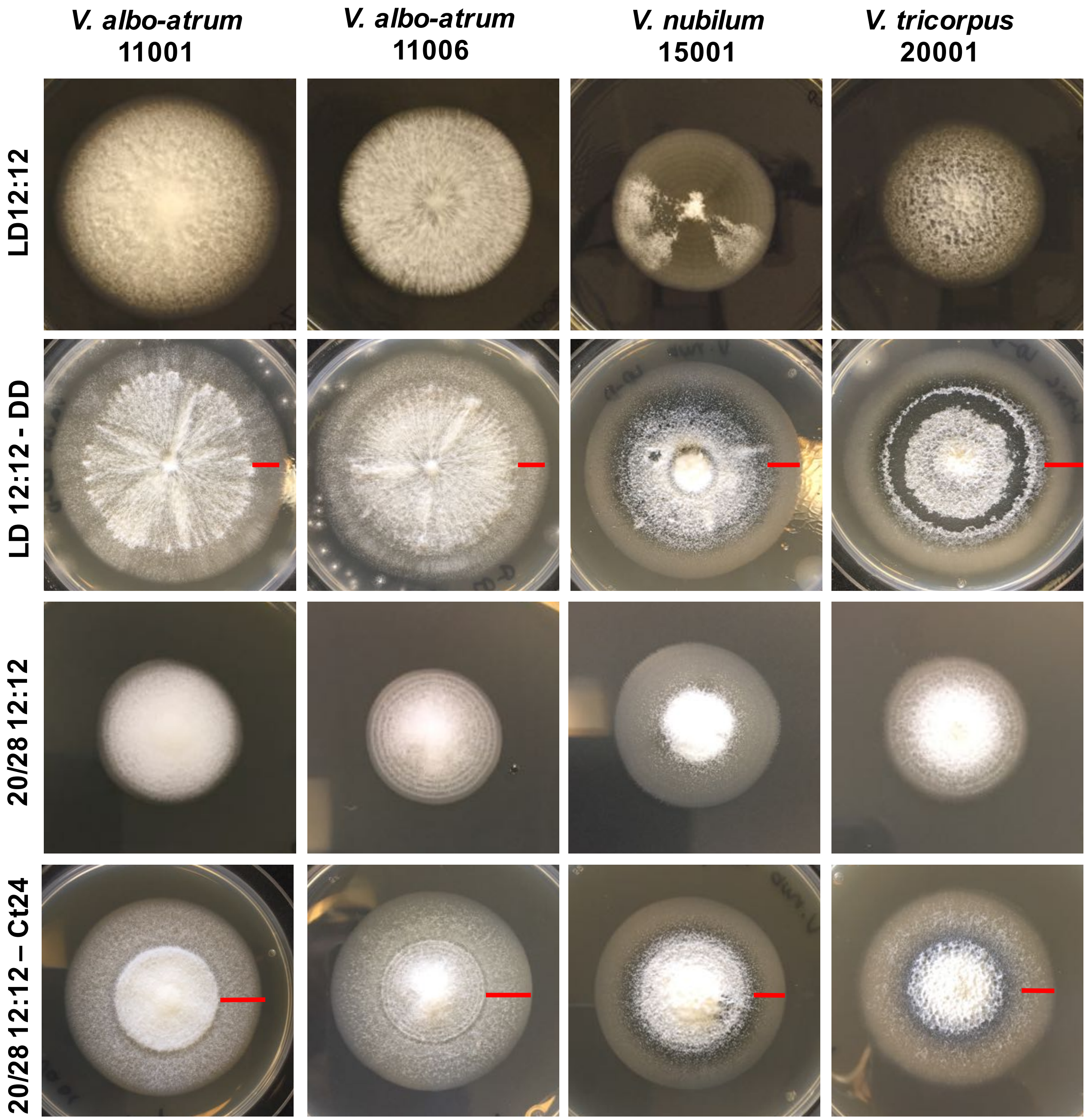

Suppl. Figure 7

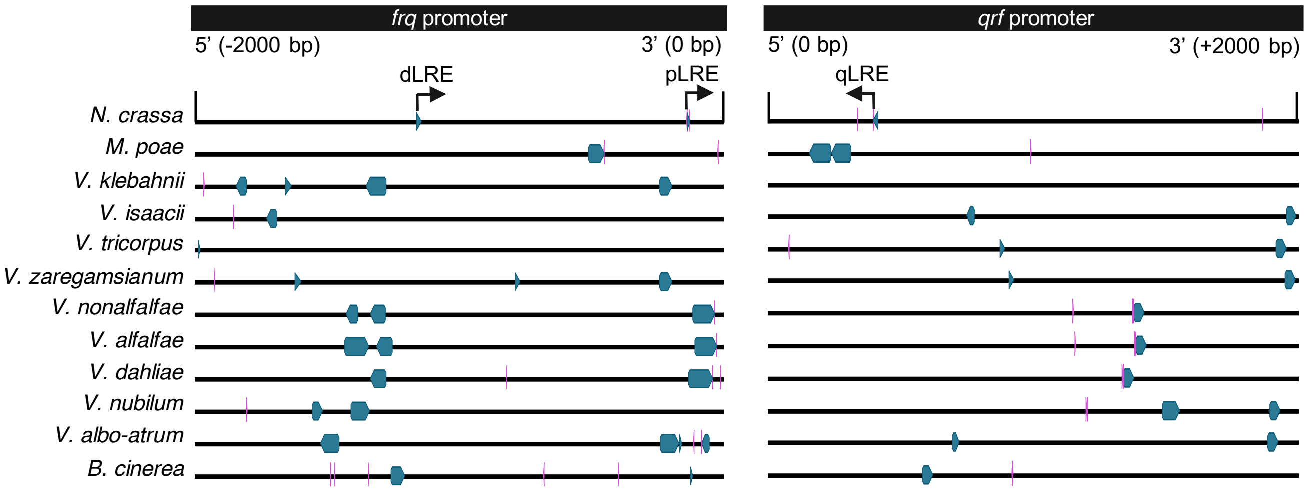

Suppl. Figure 8

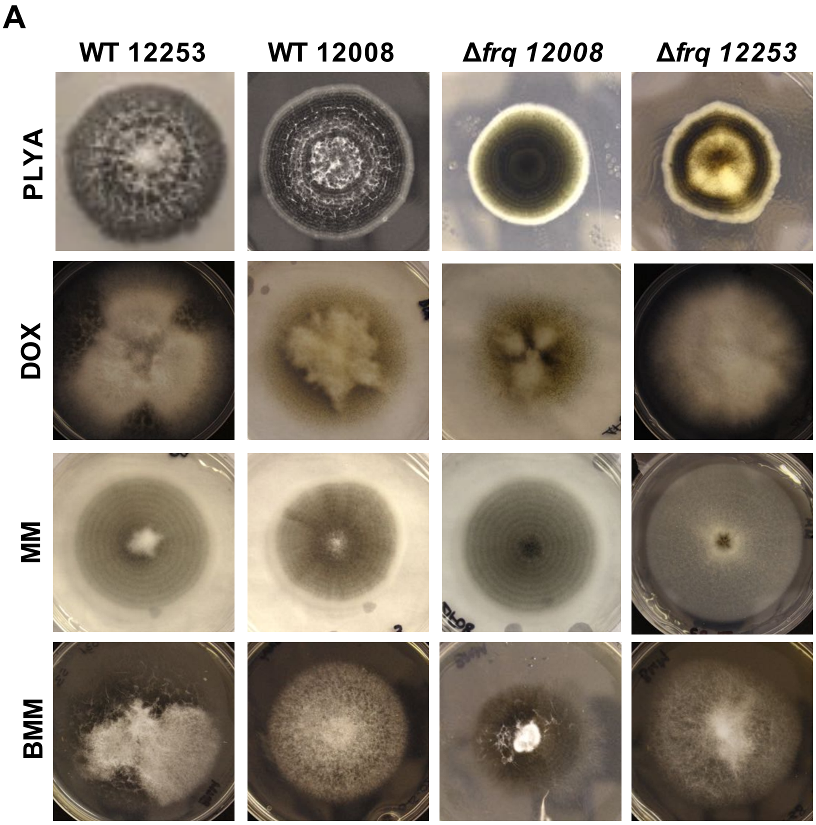

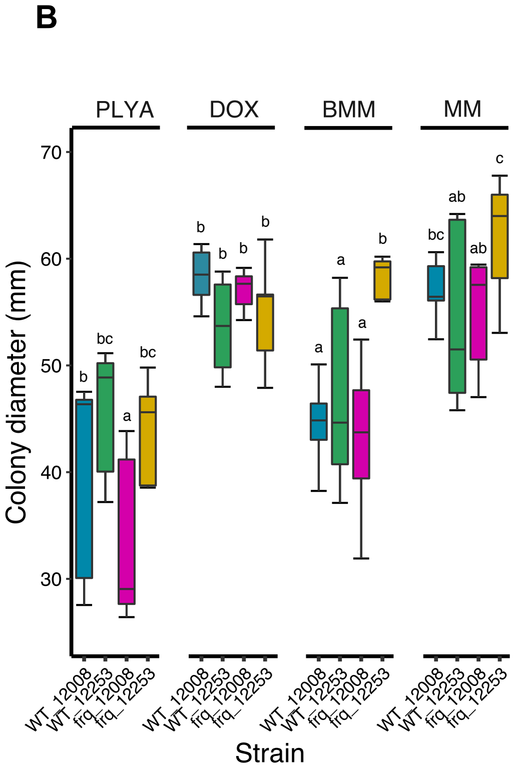

Supplement: FIGURE S1 — Generation of V. dahliaeΔfrq mutants. (A) Strategy followed to replace V. dahliae frq with hygromycin phosphotransferase. Genomic regions utilized for homologous recombination (black fragments) are shown. Black arrows symbolize primer pairs used for the validation PCR. (B) Representative gel of the validation PCRs for the correct V. dahliae knockout transformants. (C) Primer pairs utilized in PCR validation. [file Data_Sheet_2.PDF]
