# Supplementary material for: No Evidence That Homologs of Key Circadian Clock Genes Direct Circadian Programs of Development or mRNA Abundance in Verticillium dahliae
Source: Front Microbiol. 2020 Aug 28;11:1977. doi: 10.3389/fmicb.2020.01977 (PMC7493669; doi:10.3389/fmicb.2020.01977)
Supplement: TABLE S1 — List of fungal strains. ∗Unknown data is labeled as Not Available (NA). [file Data_Sheet_1.PDF]

S1 Table. List of fungal strains.

Species	Isolate	Host	Origin	Date isolated	Source
V.dahliae	12008	Strawberry	Kent, UK	1985	Laboratory stock
V.dahliae	12061	Strawberry	Lenham, UK	1986	Laboratory stock
V.dahliae	12062	Strawberry	Hampshire, UK	1965	Laboratory stock
V.dahliae	12064	Chrysanthemum	Bristol, UK	1968	Laboratory stock
V.dahliae	12065	Potato	Canada, USA	1969	Laboratory stock
V.dahliae	12067	Tomato	Dorset, UK	1971	Laboratory stock
V.dahliae	12068	Hops	UK	NA	Laboratory stock
V.dahliae	12072	Gerbera	Japan	1998	Laboratory stock
V.dahliae	12073	Chinese Cabbage	Japan	1998	Laboratory stock
V.dahliae	12086	Strawberry - petiole	Kent, UK	1998	Laboratory stock
V. dahliae	12158	Strawberry	Kent, UK	2000	Laboratory stock
V. dahliae	12161	Strawberry	Lincolnshire, UK	2000	Laboratory stock
V. dahliae	12251	Strawberry	Kent, UK	2012	Laboratory stock
V. dahliae	12252	Strawberry	Kent, UK	2012	Laboratory stock
V. dahliae	12253	Strawberry	Kent, UK	2012	Laboratory stock
V.dahliae	12213	Olive	NA	2002	Laboratory stock
V. albo-atrum	11001	Hop	Kent, UK	1987	Laboratory stock
V. albo-atrum	11006	Hop	Kent, UK	1995	Laboratory stock
V. nubilum	15001	NA*	NA	NA	Laboratory stock
V. tricorpus	20001	NA	NA	1998	Laboratory stock
V. dahliae	*Δfrq_12008*	-	-	-	This study
V. dahliae	*Δfrq_12253*	-	-	-	This study
V. dahliae	*Δwc1_12253*	-	-	-	This study
N. crassa	*30-7 bd a*	-	NA	NA	S. Crosthwaite

S2 Table. List of primers.

Organism	Target	Gene name	Name	Sequence (5' to 3')	Length (bp)	Sequence reference
V.dahliae	Housekeeping	Elongation factor alpha	EFA1-F	TACAACCCCAAGACTGTCGC	104	[112]
V.dahliae	Housekeeping		EFA1-R	TCCTTCTCCCAGCCCTTGTA		
V.dahliae	Housekeeping	Tubuline Beta Chain	VdTbc-F	TTTCCAGATCACCCACTCC	111	[49, 113]
V.dahliae	Housekeeping		VdTbc-R	ACGACCGAGAAGGTAGCC		
V.dahliae	Gene of interest	frq	VdFrq-F	GTGACGGAGAAGAGCACCAA	92	This study
V.dahliae	Gene of interest		VdFrq-R	TCGCTACTGAATCGCGTACC		
V.dahliae	Gene of interest	Wc-1	VdWc1-F	CAACACCCTGACCGAGTTCA	91	This study
V.dahliae	Gene of interest		VdWc1-R	ATTGGCGTCGAGAATCTGCA		
V.dahliae	Gene of interest	cry	Vdcry_F	GAAGGGTGGTGAGGTGGATG	177	This study
V.dahliae	Gene of interest		Vdcry_R	TTCACCATCTCCTCGTGCAC		
V.dahliae	Gene of interest	ccg16	Vdccg16_F	CCCAAGGCATCACAGTCGAA	118	This study
V.dahliae	Gene of interest		Vdccg16_R	GCACCATTGTTGTTGCCGAT		
V.dahliae	Gene of interest	vvd	Vdvvd_F	GATGTTCATGAACGGCGAGC	135	This study
V.dahliae	Gene of interest		Vdvvd_R	GTCAGCCGATCTTCTCCTCG		
N.crassa	Housekeeping	TATA binding box protein	NcTbp-F	GGTGCCAAGTCCGAAGATGA	136	[114]
N.crassa	Housekeeping		NcTbp-R	TGGGGAACTTGATGTCGCAA		
N.crassa	Housekeeping	Beta-tubulin	NcBtl-F	CCACTTCTTCATGGTCGGCT	103	[114]
N.crassa	Housekeeping		NcBtl-R	CTTGGGGTCGAACATCTGCT		
N.crassa	Gene of interest	Frequency	NcFrq-F	TCGACATCGCAGAGGAGAAA	66	[115]
N.crassa	Gene of interest		NcFrq-R	CAACGAAACCCCAGACGAGT		

	Primer pair	DNA Template	Overhang 5' - 3'	Sequence 5' - 3'	Length (bp)	Sequence reference
USER-Brick	B1-F	pRF-HU2	AAGGTTTAAU	TCACTGGCCGTCGTTTTA	2165	[56]
	B1-R		ATTTAAAGAU	CCGCGCGAGC		
	HygR1-F	pRF-HU2	ACGCAATACU	AGTCGGGGGATCCTCTAG	2480	[56]
	HygR1-R		ACTAGGTCAU	GGGCCCATCGATGATCAG		
	B2-F	pRF-HU2	ATCTTTAAAU	GGAGTGTCTTCTTCCCA	1658	[56]
	B2-R		AATACGACCU	TCGTGACTCCCTTAATTCT		
	HRFrq1-F	*V. dahliae*	AGGTCGTATU	GAGACTTCAGTAAATTGTGGTTGT	1600	This study
	HRFrq1-R		AGTATTGCGU	AGAGTCGTTGTCGTCGGG		
	HRFrq2-F	*V. dahliae*	ATGACCTAGU	GATCACATTTTGCATCTTTCGGG	1565	This study
	HRFrq2-R		ATTAAACCTU	GTCACCATGACTCGCTACAA		
Plasmid validation	B1.B2-F	pEcFrq-D1		TGTCATACCACTTGTCCGCC	461	This study
	B1.B2-R			CTGCCTGTTCCAAAGGTCCT		
	B1.B2.F1.H-F	pEcFrq-D1		ATGTTGCTGTCTCCCAGGTC	3600	This study
	B1.B2.F1.H-R			CCTATATCGCCGACATCACC		
	H.F2.B1-F	pEcFrq-D1		GTATGACCGGGTCGTTCACT	2190	This study
	H.F2.B1-R			CTGGCTGGTGGCAGGATA		
V. dahliae mutants validation	TestHygr_F	Δfrq mutants		ATTTGTGTACGCCCGACAGT	624	This study
	TestHygr_R			AGACCTGCCTGAAACCGAAC		
	TestFrq-F	Δfrq mutants		CCATCTTCGGCGCATTTGAG	563	This study
	TestFrq-R			ACTGTGAGGAATTGCTGCGA		
	FrqUS_Hygr-F	Δfrq mutants		AGTTCCACTCGTTCGCTCTG	2182	This study
	FrqUS_Hygr-R			CGCCTATATCGCCGACATCA		

S3 Table. List of significantly enriched GO terms related to biological processes in *Δfrq_12253* versus the WT_12253 strain in light and dark.

		**Δfrq vs WT in D**		**Δfrq vs WT in L**	
**GO.ID**	**Biological process term**	**UP**	**DOWN**	**UP**	**DOWN**
GO:0006412	translation	5.40E-11		2.00E-16	
GO:0006414	translational elongation	3.40E-05			
GO:0008152	metabolic process	0.022		0.01	
GO:0045039	protein import into mitochondrial inner membrane	0.024			
GO:0009306	protein secretion	0.024		0.032	
GO:0006228	UTP biosynthetic process	0.024		0.032	
GO:0006241	CTP biosynthetic process	0.024		0.032	
GO:0046836	glycolipid transport	0.024			
GO:0006183	GTP biosynthetic process	0.024		0.032	
GO:0009116	nucleoside metabolic process	0.042			
GO:0033566	gamma-tubulin complex localization			0.032	
GO:0055114	oxidation-reduction process		2.90E-05		8.20E-05
GO:0007623	circadian rhythm		0.016		0.0221
GO:0019427	acetyl-CoA biosynthetic process from acetate		0.016		
GO:0006788	heme oxidation		0.016		0.0221
GO:0009448	gamma-aminobutyric acid metabolic processes		0.031		
GO:0006537	glutamate biosynthetic process		0.032		0.0437
GO:0001682	tRNA 5'-leader removal		0.032		
GO:0009450	gamma-aminobutyric acid catabolic processes		0.032		
GO:0072488	ammonium transmembrane transport		0.047		
GO:0006817	phosphate ion transport				0.0046
GO:0042128	nitrate assimilation				0.0046
GO:0006810	transport				0.0129
GO:0042554	superoxide anion generation				0.0221
GO:0009405	pathogenesis				0.0221
GO:0006809	nitric oxide biosynthetic process				0.0221
GO:0000160	phosphorelay signal transduction system				0.042
GO:0009395	phospholipid catabolic process				0.0437
GO:0006730	one-carbon metabolic process				0.0437

S4 Table. Expression of putative core clock genes, photoreceptor- and TF-encoding genes in WT 12253 and *Δfrq_12253* in both light and dark conditions. Transcripts displaying a Log fold change (LFC) >1 were classified as pink, dark red if the LFC > 2, light green if the LFC < -1 and dark green if the LFC < -2. Yellow boxes indicate *p*- values < 0.05.

		L/D in V.d 12253		L/D in Δfrq_12253		Δfrq/WT in D		Δfrq/WT in L					
**Transcript_id**	**baseMean**	**LFC**	**p-value**	**LFC**	**p-value**	**LFC**	**p-value**	**LFC**	**p-value**	**Gene**	**Antismash**	**SM**	**Interpro**
**Clock oscillator genes**													
VDAG_JR2_Chr6g03850	2113.89	0.07	0.51	-0.21	0.10	-0.16	0.08	-0.45	0.00	*fwd-1*	cluster_63	cf_putative	F-box; WD40 repeat
VDAG_JR2_Chr2g01990	1748.38	0.17	0.23	-0.16	0.47	-0.30	0.02	-0.63	0.00	*wc-1*			Zinc finger, GATA-type
VDAG_JR2_Chr4g00070	1474.05	-0.01	0.94	-0.11	0.66	0.06	0.74	-0.04	0.85	*frh*			ATP-binding; Helicase; rRNA-processing arch, Ski2
VDAG_JR2_Chr3g10380	1236.87	-0.05	0.83	0.97	0.00	-0.53	0.00	0.49	0.01	*vvd*			LOV
VDAG_JR2_Chr7g03830	698.85	-0.10	0.47	-0.23	0.17	-0.68	0.00	-0.82	0.00	*wc-2*			Zinc finger, GATA-type
**Photoreceptors genes**													
VDAG_JR2_Chr7g03830	698.85	-0.10	0.47	-0.23	0.17	-0.68	0.00	-0.82	0.00	*wc-2*			Zinc finger, GATA-type
VDAG_JR2_Chr2g01990	1748.38	0.17	0.23	-0.16	0.47	-0.30	0.02	-0.63	0.00	*wc-1*			Zinc finger, GATA-type
VDAG_JR2_Chr3g10380	1236.87	-0.05	0.83	0.97	0.00	-0.53	0.00	0.49	0.01	*vvd*			LOV
VDAG_JR2_Chr1g22390	1602.12	0.05	0.77	-0.04	0.89	0.23	0.07	0.14	0.36	*rgs-lov*			PAS

VDAG_JR2_Chr6g00620	191.19	-0.07	0.82	0.03	0.95	-0.35	0.09	-0.25	0.29	*cry-1*	cluster_60	other	Photolyase/cryptochrome; FAD-binding
VDAG_JR2_Chr4g09150	1433.05	0.26	0.17	0.11	0.73	-0.21	0.27	-0.36	0.06	*phy*			HK; PAS; Phytochrome
VDAG_JR2_Chr7g00170	1376.59	-0.06	0.68	0.14	0.41	0.01	0.92	0.21	0.07	*phr*			DNA-photolyase; FAD-binding
VDAG_JR2_Chr1g29230	614.73	0.56	0.18	0.31	0.63	0.65	0.09	0.40	0.36	*nop-1*			G-protein glucose receptor; Rhodopsin
VDAG_JR2_Chr6g07410	386.51	0.56	0.00	0.19	0.53	-0.40	0.02	-0.77	0.00	*lov-u*			PAS
VDAG_JR2_Chr1g17210	349.67	0.99	0.00	1.11	0.00	-1.16	0.00	-1.05	0.00	*cry-dash*	cluster_8	terpene	Photolyase/cryptochrome; FAD-binding
**Transcription factors**													
VDAG_JR2_Chr1g26850	91.41	-1.03	0.00	-0.19	0.72	-0.93	0.00	-0.09	0.81				Zn2 Cys6;Fungal-specific TF
VDAG_JR2_Chr5g05720	27062.18	-1.01	0.00	-0.52	0.07	-0.81	0.00	-0.32	0.19				Zinc finger, C2H2-type
VDAG_JR2_Chr2g04990	4202.92	-1.05	0.00	-0.67	0.06	-0.35	0.20	0.03	0.93				Nucleic acid-binding proteins
VDAG_JR2_Chr1g24030	47.75	-1.23	0.00	-0.53	0.17	-0.20	0.52	0.50	0.12				Zn2 Cys6;Fungal-specific TF
VDAG_JR2_Chr5g05660	1280.98	1.33	0.00	0.75	0.01	-0.01	0.98	-0.58	0.01				Zinc finger, C2H2-type
VDAG_JR2_Chr1g23950	945.69	1.68	0.00	0.69	0.24	0.13	0.81	-0.87	0.05		cluster_14	t1pks-nrps	Zn2 Cys6
VDAG_JR2_Chr4g03300	951.19	0.51	0.01	1.09	0.00	0.35	0.06	0.93	0.00				Zn2 Cys6

VDAG_JR2_Chr6g08330	9249.77	-0.76	0.00	-0.45	0.01	-1.27	0.00	-0.95	0.00	cluster_68	cf_putative	Zinc finger, C2H2-type;Fungal TF
VDAG_JR2_Chr8g07070	412.26	-0.40	0.18	0.18	0.71	-1.13	0.00	-0.55	0.07			Zn2 Cys6
VDAG_JR2_Chr3g04200	36396.10	-0.91	0.00	-0.38	0.13	-1.11	0.00	-0.58	0.00			Zinc finger, C2H2-type
VDAG_JR2_Chr2g11050	3358.89	0.59	0.00	0.47	0.01	1.10	0.00	0.97	0.00			Zn2 Cys6
VDAG_JR2_Chr2g03990	18.20	0.77	0.11	-0.07	0.94	1.31	0.00	0.48	0.32	cluster_19	cf_putative	Fungal-specific TF
VDAG_JR2_Chr8g00200	52.88	-1.92	0.00	0.17	0.82	-1.55	0.00	0.54	0.22			C2H2 and C2HC zinc fingers
VDAG_JR2_Chr7g07100	23846.48	-1.01	0.00	-0.72	0.02	-1.11	0.00	-0.83	0.00			HLH
VDAG_JR2_Chr5g05400	1535.28	-0.24	0.12	-0.33	0.11	-0.93	0.00	-1.02	0.00			Zn2 Cys6
VDAG_JR2_Chr1g03770	112.66	0.86	0.00	0.28	0.60	-0.88	0.00	-1.47	0.00			Zn2/Cys6 DNA-binding domain
VDAG_JR2_Chr1g10470	1305.60	0.29	0.18	-0.11	0.78	-0.85	0.00	-1.25	0.00			Zn2 Cys6
VDAG_JR2_Chr4g09890	2291.00	0.17	0.38	-0.15	0.62	-0.72	0.00	-1.03	0.00	cluster_45	cf_putative	Zn2 Cys6
VDAG_JR2_Chr3g08070	3513.45	0.75	0.00	0.29	0.45	-0.67	0.00	-1.13	0.00			bZIP
VDAG_JR2_Chr1g27300	905.11	1.00	0.00	-0.05	0.87	-0.29	0.05	-1.34	0.00			Zn2 Cys6
VDAG_JR2_Chr5g00990	503.03	-0.58	0.05	0.24	0.62	0.34	0.25	1.16	0.00			Zn2 Cys6;Fungal-specific TF
VDAG_JR2_Chr7g02150	631.00	-0.30	0.15	0.37	0.16	0.43	0.02	1.10	0.00			HLH
VDAG_JR2_Chr1g22480	307.59	-0.20	0.36	0.30	0.25	0.56	0.00	1.06	0.00			Zn2 Cys6

VDAG_JR2_Chr2g07670	8272.23	-0.23	0.12	0.24	0.24	0.59	0.00	1.05	0.00			Nucleic acid-binding proteins
VDAG_JR2_Chr2g10410	99.24	-0.05	0.89	0.32	0.29	0.64	0.00	1.00	0.00			Zn2 Cys6
VDAG_JR2_Chr8g08220	276.28	-0.15	0.43	0.23	0.30	0.65	0.00	1.03	0.00			Nucleic acid-binding proteins
VDAG_JR2_Chr2g11140	4420.16	-0.41	0.12	0.16	0.73	0.72	0.00	1.28	0.00			Zinc finger, C2H2-type
VDAG_JR2_Chr2g03270	259.52	-0.57	0.08	0.37	0.44	0.77	0.01	1.72	0.00	cluster_18	cf_putative	Zn2 Cys6;Fungal-specific TF
VDAG_JR2_Chr2g12610	11872.65	0.06	0.83	0.29	0.34	0.82	0.00	1.05	0.00			Glucocorticoid receptor-like
VDAG_JR2_Chr5g10370	2513.36	-0.79	0.00	0.11	0.75	0.83	0.00	1.73	0.00			bZIP
VDAG_JR2_Chr4g07710	13092.22	0.07	0.78	0.24	0.42	0.86	0.00	1.03	0.00			Winged helix DNA-binding domain
VDAG_JR2_Chr6g06230	2733.17	0.06	0.76	0.26	0.23	0.87	0.00	1.07	0.00			Winged helix DNA-binding domain
VDAG_JR2_Chr4g11420	52.20	-0.03	0.95	0.20	0.68	0.90	0.00	1.13	0.00	cluster_48	cf_putative	Zn2 Cys6
VDAG_JR2_Chr1g03460	762.45	0.14	0.48	0.30	0.18	0.93	0.00	1.09	0.00			Homeodomain-like
VDAG_JR2_Chr4g10010	79.48	-0.24	0.51	-0.07	0.90	0.98	0.00	1.14	0.00	cluster_45	cf_putative	MADS-box
VDAG_JR2_Chr3g06100	289.98	-0.15	0.68	0.48	0.18	0.98	0.00	1.61	0.00			Zn2 Cys6;Fungal-specific TF
VDAG_JR2_Chr8g01630	439.62	-1.26	0.00	0.42	0.31	-0.59	0.03	1.09	0.00			Zinc finger, C2H2-type;Fungal TF
VDAG_JR2_Chr5g01890	247.82	1.56	0.00	-0.16	0.75	-0.49	0.07	-2.22	0.00			Zn2 Cys6;Fungal-specific TF

VDAG_JR2_Chr5g10380	25.61	-0.26	0.62	1.14	0.01	0.67	0.08	2.07	0.00			Zn2 Cys6
VDAG_JR2_Chr2g13070	264.38	0.00	0.99	-0.12	0.77	-1.53	0.00	-1.66	0.00	cluster_27	cf_putative	Zn2/Cys6
VDAG_JR2_Chr6g07650	2339.84	-0.12	0.56	-0.20	0.47	-1.13	0.00	-1.21	0.00			C2H2
VDAG_JR2_Chr3g07150	3982.06	-0.15	0.44	-0.20	0.47	-1.02	0.00	-1.06	0.00			Homeodomain-like
VDAG_JR2_Chr1g05850	11542.15	0.12	0.61	0.13	0.71	1.00	0.00	1.01	0.00			Nucleic acid-binding proteins
VDAG_JR2_Chr4g03340	253.43	-0.02	0.94	0.18	0.54	1.08	0.00	1.29	0.00			Zn2 Cys6
VDAG_JR2_Chr2g10850	51.00	0.16	0.65	0.27	0.49	1.19	0.00	1.30	0.00			Zn2 Cys6
VDAG_JR2_Chr2g00520	69.36	-0.15	0.77	0.87	0.08	1.29	0.00	2.32	0.00	cluster_17	t1pks	Winged helix DNA-binding domain
VDAG_JR2_Chr8g11340	2413.49	0.04	0.91	-0.11	0.80	1.54	0.00	1.40	0.00			Zinc finger, C2H2-type
VDAG_JR2_Chr2g00510	2.81	-0.27	0.65	0.30	NA	1.79	0.00	2.35	0.00	cluster_17	t1pks	Homeodomain-like
VDAG_JR2_Chr7g10270	2637.45	0.28	0.30	0.10	0.83	2.35	0.00	2.17	0.00	cluster_78	nrps	bZIP
VDAG_JR2_Chr3g13030	42.82	1.35	0.00	0.67	0.19	-1.10	0.00	-1.78	0.00			Zn2 Cys6

S5 Table. Most differentially expressed genes in *Δfrq_12253*. Transcripts displaying a Log fold change (LFC) >1 were classified as pink, dark red if the LFC > 2, light green if the LFC < -1 and dark green if the LFC < -2. Yellow boxes indicate *p*- values < 0.05.

Transcript_id	baseMean	L/D in V.d 12253 LFC	L/D in V.d 12253 p-value	L/D in Δfrq_12253 LFC	L/D in Δfrq_12253 p-value	Δfrq/WT in D LFC	Δfrq/WT in D p-value	Δfrq/WT in L LFC	Δfrq/WT in L p-value	Antismash	SM	TFs	Interpro
**Top Up-regulated genes**													
VDAG_JR2_Chr7g10300	121.36	0.60	0.10	0.16	0.69	3.89	0.00	3.45	0.00	cluster_78	nrps		hypothetical protein
VDAG_JR2_Chr4g11240	55.89	0.05	0.94	1.20	0.05	1.98	0.00	3.13	0.00	cluster_47	t1pks		tubulin-tyrosine ligase
VDAG_JR2_Chr7g10290	151.84	0.59	0.16	0.21	0.73	3.48	0.00	3.09	0.00	cluster_78	nrps		hypothetical protein
VDAG_JR2_Chr7g02720	1334.29	-0.08	0.87	0.53	0.29	2.38	0.00	2.99	0.00				Integral membrane protein
VDAG_JR2_Chr2g00550	10.31	0.39	0.51	1.34	0.02	1.92	0.00	2.87	0.00	cluster_17	t1pks		FAD binding domain-containing protein
VDAG_JR2_Chr8g11360	452.42	-0.26	0.47	0.45	0.29	2.14	0.00	2.86	0.00				hypothetical protein
VDAG_JR2_Chr2g00500	5.95	0.08	0.92	0.69	0.31	2.24	0.00	2.85	0.00	cluster_17	t1pks		hypothetical protein
VDAG_JR2_Chr8g11370	1056.59	-0.17	0.65	0.49	0.25	2.09	0.00	2.76	0.00				S-adenosyl-L-methionine-dependent methyltransferase
VDAG_JR2_Chr2g00490	58.35	0.63	0.26	0.87	0.17	2.43	0.00	2.67	0.00	cluster_17	t1pks		serine 3-dehydrogenase

VDAG_JR2_Chr7g10260	317.52	0.20	0.48	0.79	0.00	1.90	0.00	2.49	0.00	cluster_78	nrps		hypothetical protein
VDAG_JR2_Chr7g02710	2265.56	-0.80	0.00	0.12	0.81	1.49	0.00	2.42	0.00				Cytochrome
VDAG_JR2_Chr1g24880	24.81	0.20	0.73	-0.41	0.52	2.99	0.00	2.38	0.00				cerato-ulmin hydrophobin
VDAG_JR2_Chr2g00510	2.81	-0.27	0.65	0.30	NA	1.79	0.00	2.35	0.00	cluster_17	t1pks	Homeodomain-like	Myb-transcription protein
VDAG_JR2_Chr1g23290	36.88	-0.67	0.08	-0.78	0.06	2.43	0.00	2.33	0.00	cluster_13	cf_putative		hypothetical protein
VDAG_JR2_Chr2g00520	69.36	-0.15	0.77	0.87	0.08	1.29	0.00	2.32	0.00	cluster_17	t1pks	Winged helix	S-adenosyl-L-methionine-dependent methyltransferase
VDAG_JR2_Chr5g05550	22.44	-0.70	0.06	0.66	0.10	0.94	0.00	2.30	0.00				hypothetical protein
VDAG_JR2_Chr8g02470	55.11	0.16	0.77	2.07	0.00	0.38	0.40	2.28	0.00				mannose-6-phosphate isomerase
VDAG_JR2_Chr2g02910	43.72	1.45	0.00	2.67	0.00	1.01	0.02	2.22	0.00				hypothetical protein
VDAG_JR2_Chr1g27430	31.46	-0.73	0.05	0.38	0.40	1.11	0.00	2.22	0.00				hypothetical protein
VDAG_JR2_Chr8g11350	1851.53	-0.11	0.80	0.01	0.99	2.06	0.00	2.18	0.00				Cytochrome
VDAG_JR2_Chr7g10270	2637.45	0.28	0.30	0.10	0.83	2.35	0.00	2.17	0.00	cluster_78	nrps	bZIP	bZIP transcription factor

VDAG_JR2_Chr8g11140	201.79	-0.32	0.39	0.48	0.28	1.37	0.00	2.17	0.00				hypothetical protein
VDAG_JR2_Chr7g04960	100.68	-0.70	0.11	-0.47	0.44	1.93	0.00	2.16	0.00				NADP-dependent oxidoreductase domain-containing
VDAG_JR2_Chr2g00540	97.06	-0.13	0.80	1.13	0.02	0.87	0.02	2.14	0.00	cluster_17	t1pks		Alpha/Beta hydrolase
VDAG_JR2_Chr8g09650	122.03	0.79	0.06	0.61	0.27	2.26	0.00	2.09	0.00				Glycoside hydrolase family
VDAG_JR2_Chr5g10380	25.61	-0.26	0.62	1.14	0.01	0.67	0.08	2.07	0.00			Zn2 Cys6	Zn2-Cys6 transcription factor
VDAG_JR2_Chr4g03070	89.63	2.26	0.00	1.51	0.00	2.70	0.00	1.95	0.00				Stress-induced protein, KGG
VDAG_JR2_Chr6g03720	31.42	1.08	0.02	0.56	0.37	2.44	0.00	1.92	0.00	cluster_63	cf_putative		hypothetical protein
VDAG_JR2_Chr1g05450	20.37	0.56	0.32	-0.19	0.81	2.24	0.00	1.49	0.00	cluster_3	cf_putative		Integral membrane protein
VDAG_JR2_Chr6g09180	468.24	1.65	0.00	0.21	0.79	2.01	0.00	0.57	0.24	cluster_70	cf_putative		isoamyl alcohol oxidase
VDAG_JR2_Chr4g10180	840.55	0.56	0.33	-1.17	0.05	2.04	0.00	0.32	0.58				Endonuclease/Exonuclease/phosphatase
**Top Down-regulated genes**													
VDAG_JR2_Chr1g23880	410.36	4.27	0.00	0.46	0.53	0.34	0.53	-3.46	0.00	cluster_14	t1pks-nrps		lovastatin nonaketide synthase

VDAG_JR2_Chr1g23930	223.91	3.76	0.00	0.75	0.25	-0.01	1.00	-3.02	0.00	cluster_14	t1pks-nrps	TOXD protein
VDAG_JR2_Chr1g23890	459.89	3.58	0.00	0.60	0.36	-0.01	0.99	-2.99	0.00	cluster_14	t1pks-nrps	hypothetical protein
VDAG_JR2_Chr8g09780	57.25	1.70	0.00	-0.20	0.81	-1.07	0.01	-2.97	0.00			hypothetical protein
VDAG_JR2_Chr1g23910	116.12	4.13	0.00	1.16	0.05	0.04	0.95	-2.93	0.00	cluster_14	t1pks-nrps	hypothetical protein
VDAG_JR2_Chr1g23920	114.15	3.40	0.00	0.47	0.50	0.08	0.91	-2.86	0.00	cluster_14	t1pks-nrps	Alpha/Beta hydrolase
VDAG_JR2_Chr1g23940	625.82	3.36	0.00	0.69	0.30	0.10	0.88	-2.58	0.00	cluster_14	t1pks-nrps	hypothetical protein
VDAG_JR2_Chr2g03470	5765.15	0.98	0.01	-0.19	0.78	-1.37	0.00	-2.54	0.00			cytochrome P450 monooxygenase
VDAG_JR2_Chr8g09260	36.24	1.08	0.02	0.29	0.71	-1.66	0.00	-2.44	0.00			hypothetical protein
VDAG_JR2_Chr6g03670	1016.68	-1.99	0.00	-0.15	0.82	-4.27	0.00	-2.44	0.00	cluster_63	cf_putative	hypothetical protein
VDAG_JR2_Chr1g22700	4387.46	0.46	0.07	-0.34	0.37	-1.58	0.00	-2.38	0.00			hypothetical protein

VDAG_JR2_Chr4g04340	2563.03	-1.19	0.00	0.37	0.55	-3.87	0.00	-2.32	0.00				hypothetical protein
VDAG_JR2_Chr6g04130	796.94	0.58	0.11	0.09	0.91	-1.79	0.00	-2.28	0.00				hypothetical protein
VDAG_JR2_Chr4g11840	41.01	1.10	0.00	-0.01	0.99	-1.15	0.00	-2.27	0.00				hypothetical protein
VDAG_JR2_Chr5g01890	247.82	1.56	0.00	-0.16	0.75	-0.49	0.07	-2.22	0.00			Zn2/Cys6-Fungal TF	Zn2-Cys6 transcription factor
VDAG_JR2_Chr6g08940	153.16	1.22	0.01	0.42	0.56	-1.38	0.00	-2.19	0.00	cluster_69	cf_putative		cytochrome P450
VDAG_JR2_Chr8g06660	13626.04	0.00	0.99	-0.68	0.06	-1.45	0.00	-2.14	0.00				Ammonium/urea transporter
VDAG_JR2_Chr8g09770	50.00	1.05	0.01	-0.36	0.58	-0.72	0.07	-2.13	0.00				hypothetical protein
VDAG_JR2_Chr1g22660	1015.42	0.87	0.00	0.06	0.92	-1.23	0.00	-2.04	0.00				UDP-glucose 4-epimerase
VDAG_JR2_Chr1g01960	4976.56	-0.01	0.98	0.77	0.00	-2.82	0.00	-2.04	0.00				Frequency clock protein
VDAG_JR2_Chr1g09750	2398.82	0.84	0.00	-0.04	0.94	-1.16	0.00	-2.03	0.00				cholesterol oxidase

VDAG_JR2_Chr6g06730	959.89	0.33	0.59	-1.68	0.00	0.01	0.99	-2.01	0.00	Protein of unknown function
VDAG_JR2_Chr3g06750	104.25	-1.28	0.01	0.30	0.71	-2.77	0.00	-1.20	0.01	hypothetical protein
VDAG_JR2_Chr5g10060	42.49	-0.46	0.39	0.17	0.84	-2.30	0.00	-1.67	0.00	hypothetical protein
VDAG_JR2_Chr1g09640	1869.91	-0.26	0.53	0.54	0.28	-2.02	0.00	-1.22	0.00	Integral membrane protein
